# Oral administration of *Akkermansia muciniphila* elevates systemic antiaging and anticancer metabolites

**DOI:** 10.18632/aging.202739

**Published:** 2021-03-02

**Authors:** Claudia Grajeda-Iglesias, Sylvère Durand, Romain Daillère, Kristina Iribarren, Fabien Lemaitre, Lisa Derosa, Fanny Aprahamian, Noélie Bossut, Nitharsshini Nirmalathasan, Frank Madeo, Laurence Zitvogel, Guido Kroemer

**Affiliations:** 1 Gustave Roussy Comprehensive Cancer Institute, Villejuif, France; 2 Centre de Recherche des Cordeliers, Equipe labellisée Par la Ligue Contre le Cancer, Université de Paris, Sorbonne Université, Inserm U1138, Institut Universitaire de France, Paris, France; 3 Metabolomics and Cell Biology Platforms, Gustave Roussy Cancer Center, Université Paris Saclay, Villejuif, France; 4 EverImmune, Villejuif, France; 5 Institute of Molecular Biosciences, NAWI Graz, University of Graz, Graz, Austria; 6 BioTechMed-Graz, Graz, Austria; 7 Field of Excellence BioHealth, University of Graz, Graz, Austria; 8 Inserm U1015, Villejuif, France; 9 Center of Clinical Investigations in Biotherapies of Cancer (CICBT) 1428, Villejuif, France; 10 Faculty of Medicine, Université Paris Saclay, Le Kremlin-Bicêtre, France; 11 Pôle De Biologie, Hôpital Européen Georges Pompidou, AP-HP, Paris, France; 12 Suzhou Institute for Systems Medicine, Chinese Academy of Medical Sciences, Suzhou, China; 13 Karolinska Institute, Department of Women’s and Children’s Health, Karolinska University Hospital, Stockholm, Sweden

**Keywords:** *Akkermansia muciniphila*, microbiota, metabolomics, polyamines, fecal microbial transplantation

## Abstract

The presence of *Akkermansia muciniphila* (Akk) in the human gut is associated with good health, leanness and fitness. Mouse experimentation has demonstrated positive effects for Akk, which counteracts aging, mediates antiobesity and antidiabetic effects, dampens inflammation and improves anticancer immunosurveillance. Clinical trials have confirmed antidiabetic effects for Akk. Here, we investigated the time-dependent effects of oral administration of Akk (which was live or pasteurized) and other bacteria to mice on the metabolome of the ileum, colon, liver and blood plasma. Metabolomics was performed by a combination of chromatographic and mass spectrometric methods, yielding a total of 1.637.227 measurements. Akk had major effects on metabolism, causing an increase in spermidine and other polyamines in the gut and in the liver. Pasteurized Akk (Akk-past) was more efficient than live Akk in elevating the intestinal concentrations of polyamines, short-chain fatty acids, 2-hydroxybutyrate, as well multiple bile acids, which also increased in the circulation. All these metabolites have previously been associated with human health, providing a biochemical basis for the beneficial effects of Akk.

## INTRODUCTION

The intestinal microbiota plays a primordial role in human physiology and pathology [1, 2]. Indeed, the human body must be conceived as a meta-organism composed by human cells as well as an overwhelming majority of microbes in the form of phages, bacteria, archaea and eukaryotes that colonize all exterior and interior body surfaces, in particular the gastrointestinal tract. Thus, the organisms composing the gut flora outnumber human cells by a factor of 10 as far as the number of cells is concerned, and by a factor of 100 if the number of genes encoded by the host and is inhabitants is calculated [3].

The transition of health to disease is often accompanied by alterations in the composition of the intestinal microbiota that shifts from a normal state (eubiosis) to a pathological state (dysbiosis). Within a complex ecosystem, such shifts cannot be explained in terms of simple linear cause-effect relationships. Rather, it appears that many components of the system, both in the host and in the gut flora, are simultaneously impacted, causing alterations in gut permeability as well as a series of disease-associated features in the host (with inter alia an increase in systemic inflammation, metabolic syndrome, reduced immune responses, and a decrease in organismal fitness) and in the microbiota (with a loss of overall diversity, a disproportionate expansion of pathogenic species and a depletion of health-associated taxa) [4–7].

Notwithstanding these complexities, the transfer of the intestinal flora by fecal microbial transplantation (FMT) from humans to mice has established the causal involvement of intestinal dysbiosis in some diseases. For example, FMT from obese persons into mice favors excessive weight gain and diabetes in the latter [8, 9]. Similarly, the transfer of feces from cancer patients that fail to respond to immunotherapy with immune checkpoint inhibitors into mice transmits subsequent immunotherapeutic failure to the rodents [10, 11]. Thus, anticancer immunocompetence can be transferred across species barriers from one host to another by FMT.

The aforementioned discoveries have placed the intestinal microbiota in the limelight of scientific research, spurring attempts to identify individual bacterial species or consortia of several microbes that have a positive impact on health. One prominent bacterium that has wide pro-health effects is *Akkermansia muciniphila* (Akk). Akk is epidemiologically associated with the consumption of health-related food items, leanness, exercise, fitness and healthy aging [7, 10, 12–16]. Its transfer into short-lived mouse strains extends longevity, supporting that Akk has an antiaging effect [17]. Preclinical experimentation supporting its antiobesity and antidiabetic effects associated with a modulation of the urinary metabolome [18] has been validated by a successful clinical trial [19]. In mice, Akk can increase the systemic concentration of anti-inflammatory factors such as α-tocopherol and β-sitosterol [20], and stimulates anticancer immune responses in the context of immunotherapy targeting the PD-1/PD-L1 interaction [10]. Mechanistically, it is a matter of debate whether Akk has to be alive to achieve these effects or whether it can be pasteurized [19]. A heat-resistant protein produced by Akk has been shown to mediate antiobesity and antidiabetic effects through the activation of Toll-like receptors 2 and 4 [18]. However, the detailed metabolic effects of Akk have not been studied in detail.

Intrigued by these observations, we decided to investigate the impact of Akk on metabolism in an unbiased fashion, by means of mass spectrometric metabolomics. For this, we transferred different bacteria, as well as human feces alone or together with Akk into mice and performed metabolomics analyses of the ileal and colic content as well as the liver and the plasma. Here, we report the metabolic effects of live (Akk) and pasteurized Akk (Akk-past) on these compartments.

## RESULTS

### Experimental design

**Figure 1:**
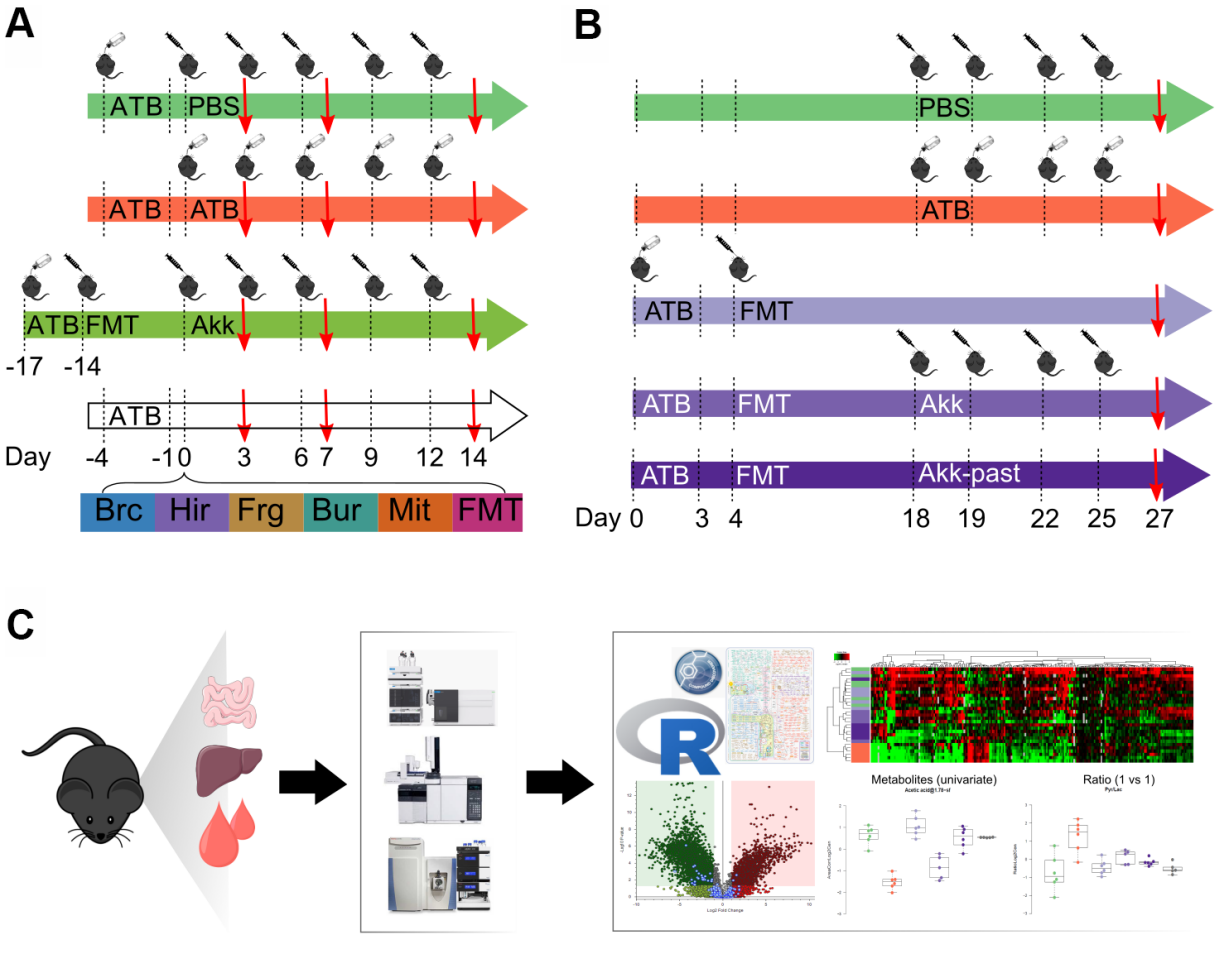
**Schematic view of the experimental design.** (**A**) Several commensals in mono-associated mice previously decontaminated by broad spectrum antibiotics (ATB) were administered by oral gavage. *Akkermansia muciniphila* (Akk) was administered by oral gavage to mice previously decontaminated by broad spectrum ATB and transplanted with human fecal material (FMT). Continuous ATB was administrated in the drinking water of the animals. (**B**) Mice previously decontaminated by broad spectrum ATB and FMT-treated, received Akk or the pasteurized form of Akk (Akk-past) by oral gavage (versus continuous FMT or ATB). PBS was administered as control (in both, (**A**, **B**) designs). (**C**) General methodology for metabolomics. The impact of the different treatments on the local (ileal, colon) and distal (liver, plasma) metabolome was evaluated using gas and liquid chromatography coupled to mass spectrometry allowing to detect a broad range of targeted and untargeted metabolites. Metabolite extraction was performed on the different organs collected from mice at the experiment day marked with a red arrow in (**A**, **B**). Extracts were processed and analyzed by liquid- and gas-chromatography coupled to mass spectrometry. Data was merged and analyzed using the GRmeta package in R or a built-in software, Compound Discoverer, for untargeted metabolomics. ATB, antibiotics; PBS, phosphate buffer saline; Brc, *Burkholderia cepacia*; Hir, *Enterococcus hirae*; Frg, *Bacteroides fragilis*; Bur, *Burkholderia* sp.; Mit, *Catenibacterium mitsuokella*; FMT, fecal microbiota transplant; Akk, *Akkermansia muciniphila*; Akk-past, pasteurized *Akkermansia muciniphila*.

In this study, mice were subjected to a defined sequence of interventions involving sham gavage with phosphate buffered saline (PBS), oral administration of broad-spectrum antibiotics (ATB, a combination of ampicillin, streptomycin and colistin), FMT from cancer patients and gavage of a series of distinct bacterial species. These species were selected because they improve the anticancer effects of cycloheximide-based chemotherapy, as true for *Burkholderia cepacia* (Brc) and *Enterococcus hirae* (Hir) [21, 22]. *Bacteroides fragilis* (Frg) was chosen because it improves chemotherapy with oxaliplatin and immunotherapy with CTLA-4 blockade [23, 24]. In addition, we included *Burkholderia* sp. (Bur) and *Catenibacterium mitsuokella* (Mit) as controls. These species were administered after ATB conditioning without prior FMT, allowing for the spontaneous recovery of the gut microflora. In contrast, since Akk (a human gastrointestinal mucin-loving bacterium) requires the presence of other bacteria to achieve efficient colonization [25], Akk was gavaged to mice that had previously received a FMT from a cancer patient that had not responded to immunotherapy and whose stools had been screened for absence of Akk. In a first experiment, only live Akk was given to mice, comparing its effects to that of other bacteria or FMT alone over time on days 3, 7 and 14 after discontinuation of ATB (Figure 1A). In a second experiment (Figure 1B), live Akk was compared to Akk-past, based on the observation that pasteurization actually does not destroy the antiobesity and antidiabetic effects of Akk [18, 19]. This experiment was designed to characterize long-term effects of Akk versus Akk-past with one single time point (day 27).

Both experiments, which involved a minimum of 6 mice per group and time point (total 245 mice) were followed by the recovery of ileal and colonic content as well as that of liver and plasma. These samples were then subjected to metabolite extraction [26], optional chemical derivatization, different types of (gas or liquid) chromatography and mass-spectrometric identification of metabolites, either in a targeted mode (in which each metabolite is identified based on its chromatographic characteristics coupled to its exact mass) or in an untargeted mode (in which each metabolite is identified as a discernible peak with a defined mass). After analysis of the samples on three different mass spectrometers, the data were subjected to R-based informatics treatments to combine results obtained by different methods into single files (one for targeted and one for untargeted results for each sample) and bioinformatics analysis (Figure 1C). In total, 1.637.227 mass spectrometric measurements were performed in this study.

### A gradient of microbial effects from the intestine to the circulation

**Figure 2:**
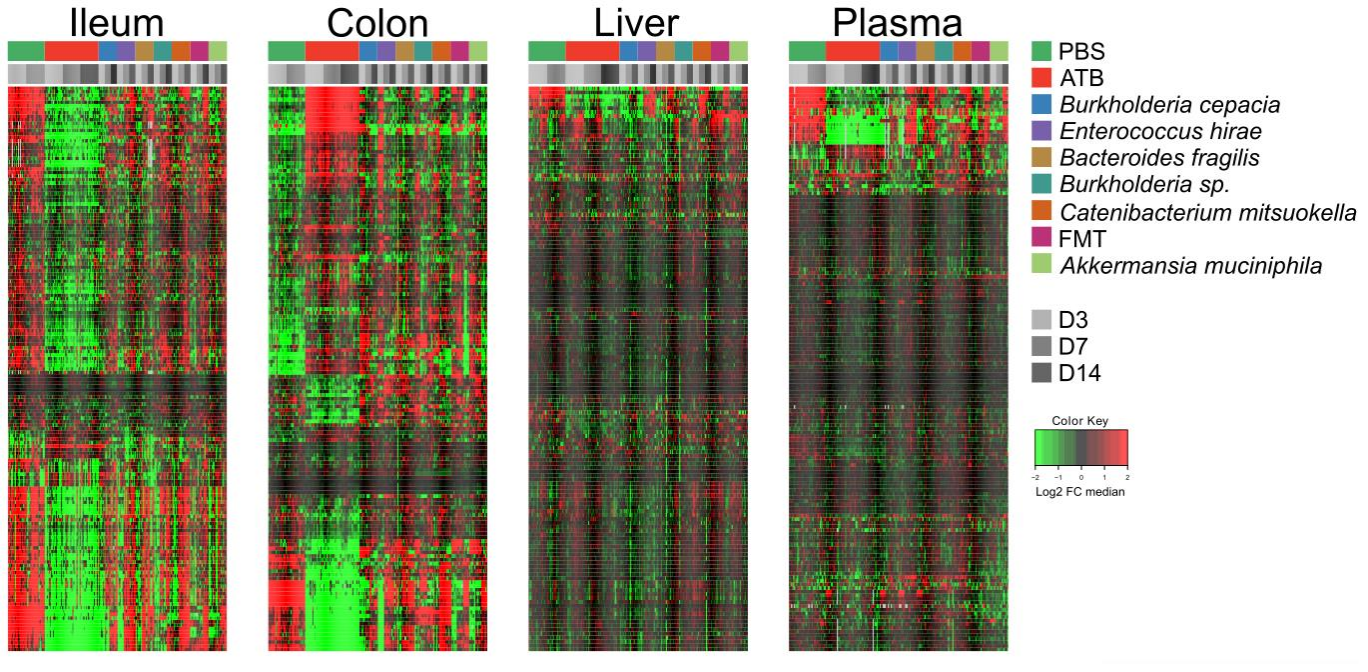
**Targeted metabolomics analysis was performed on the extracts from ileum, colon, liver, and plasma samples from mice receiving oral gavages with several commensals or PBS (control), or continuous ATB, at days 3, 7 and 14 after the first oral gavage.** Changes in metabolites relative abundance are illustrated. Ileum and colon showed the strongest treatment-dependent metabolites variations. Hierarchical clustering (euclidean distance, ward linkage method) of the metabolite abundance is shown. Note that Supplementary Figures 1, 2, 3 and 5 provide the names of each of the metabolites, for each of the different matrices. The purpose of this figure is to allow for a direct comparison of the amplitude of the metabolic effects of manipulation of the microbiota. ATB, antibiotics; PBS, phosphate buffer saline; FMT, fecal microbiota transplant; FC, fold change.

In the first experiment (Figure 1A), continuous antibiotic (ATB) treatment caused a massive depletion of ileal metabolites (as indicated by the green color in the heatmap) that was manifest on days 3, 7 and 14 post-ATB (Figure 2 and Supplementary Figure 1 and Supplementary Table 1), supporting the importance of the ileal microbiota for the breakdown of nutrients into small molecules. Thus, more than half of the metabolites in the small intestine were significantly reduced in their abundance after ATB treatment. This strong ATB effect was also observed for the colic content, in which many metabolites were reduced while others including several monosaccharides and amino acids were increased in their abundance (as indicated by the red color in the heatmap, Figure 2 and Supplementary Figure 2 and Supplementary Table 2). Gavage with individual bacteria (Brc, Bur, Frg, Hir, Mit) or FMT, alone or with Akk upon ATB discontinuation gradually corrected the ATB-induced depletion of ileal metabolites, as well as the shifts in colonic metabolism, over time, thus allowing the ileal and colic metabolomes to recover to a state that resembles the basal state (represented by the PBS control). Very similar tendencies were observed for the hepatic and plasma metabolomes, though with the important difference that the concentration of most metabolites remained close-to-unaltered and only a minority decreased (and rarely increased) upon ATB treatment (Supplementary Figure 3). Again, the ATB-induced changes in the hepatic and plasma metabolomes were reset to close-to-normal levels by reconstitution of the intestinal microbiota (Figure 2 and Supplementary Figures 4, 5 and Supplementary Tables 3, 4).

In conclusion, the composition of the hepatic and circulating metabolomes is less affected by ATB-mediated sterilization of the gut than the ileal and colic metabolome, confirming that the internal milieu is protected against external perturbations. The effects of individual bacteria on this system appear discrete.

### *Akkermansia muciniphila* (Akk)-specific effects on metabolism

**Figure 3:**
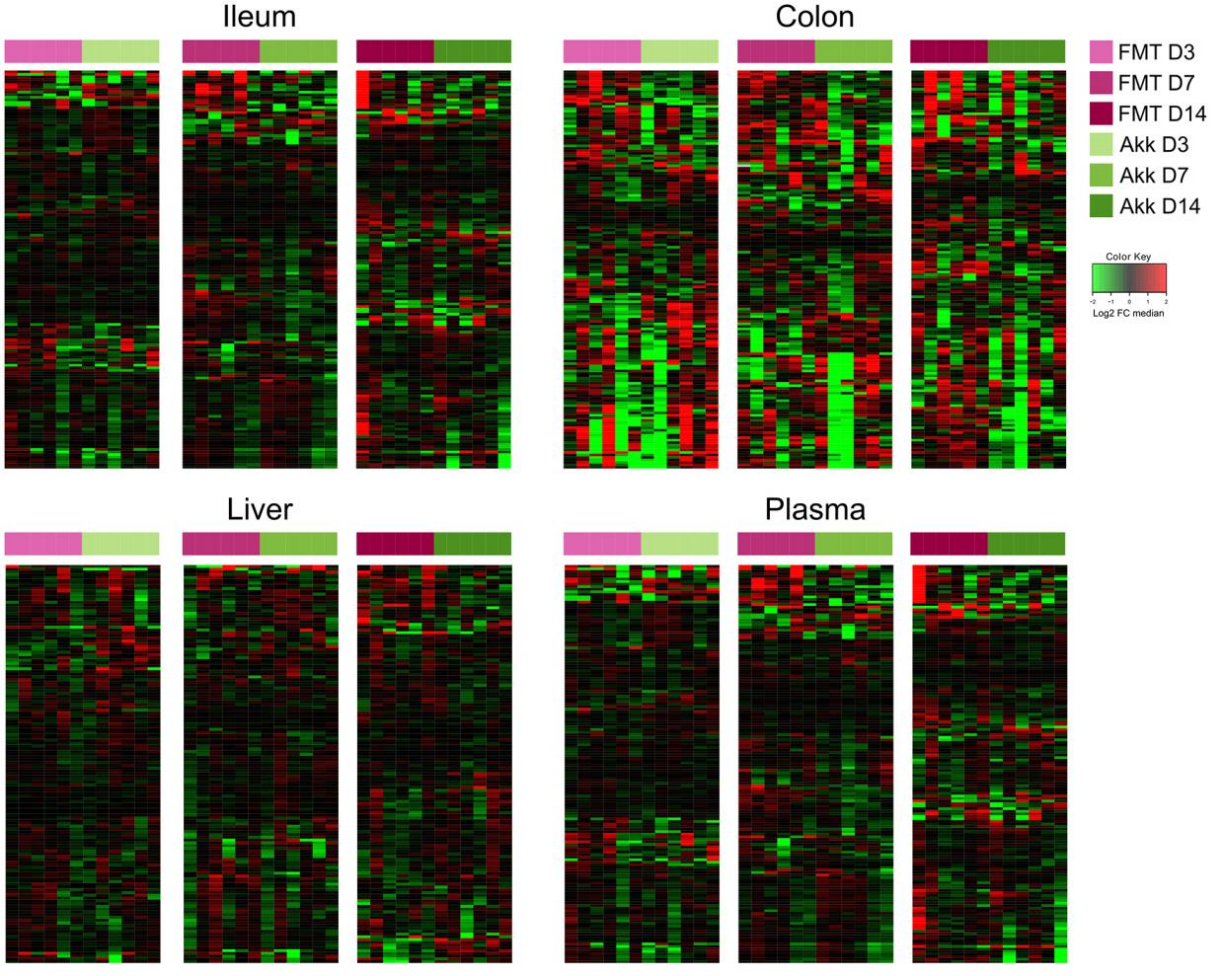
**Ileum, colon, liver, and plasma targeted metabolites relative abundance variations in mice treated with FMT or Akk at days 3, 7 and 14 after the first oral gavage.** Hierarchical clustering (euclidean distance, ward linkage method) of the metabolite abundance is shown. Note that Supplementary Figures 6 to 9 provide the names of each of the metabolites, for each of the different matrices. The purpose of this figure is to allow for a direct comparison of the amplitude of the metabolic effects of manipulation of the microbiota. FMT, fecal microbiota transplant; Akk, Akkermansia muciniphila; FC, fold change.

**Figure 4:**
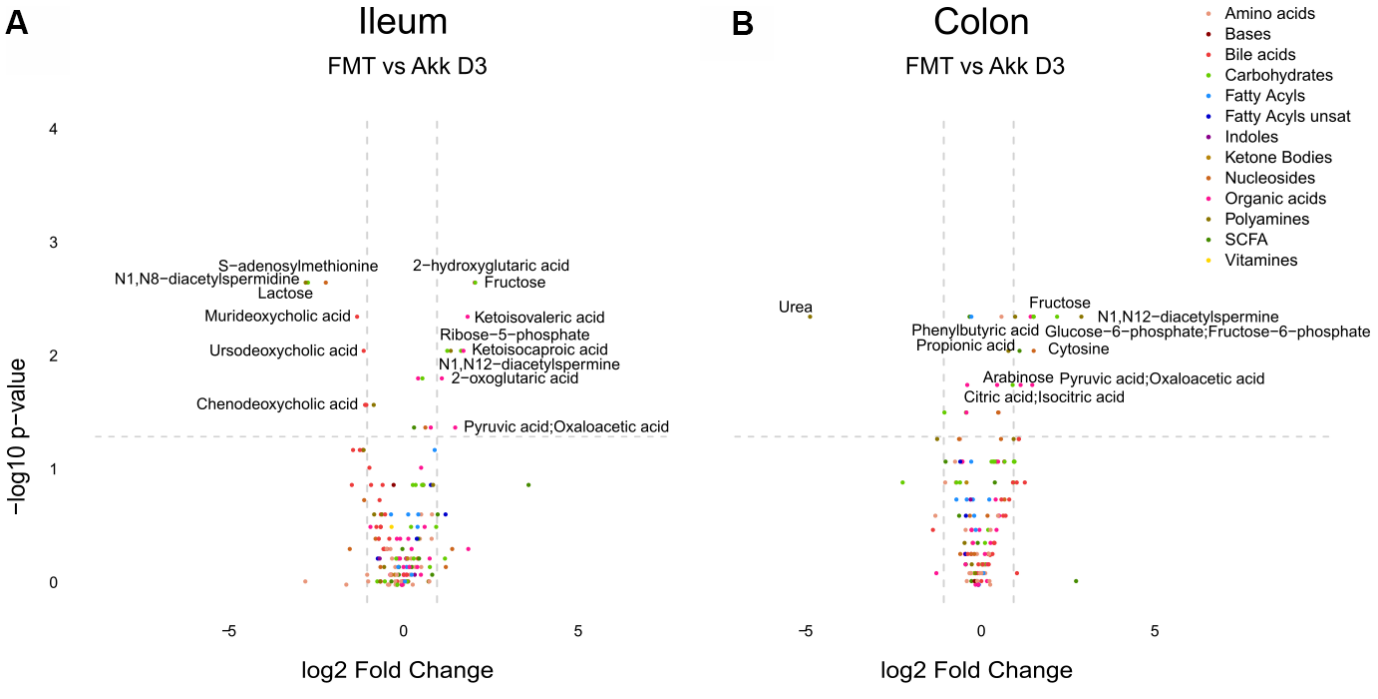
Differential metabolite identification in samples from ileum (**A**), or colon (**B**) from mice receiving FMT or Akk at day 3 after the first oral gavage. The horizontal dashed gray line shows where *p*=0.05 with points above being metabolites with significantly different relative abundance (*p*<0.05). The vertical dashed gray lines correspond to FC=1. Targeted metabolites that display both large magnitude FC and higher statistical difference (-log10 of *p* value) are annotated. Families of metabolites are grouped by colors.

In the next step, we compared the effects of Akk plus FMT with those of FMT alone on the ileal, colic hepatic and circulating metabolomes, at 3, 7 and 14 days after discontinuation of the initial ATB treatment. In this comparison (Akk+FMT versus FMT) rather discrete effects were observed by targeted metabolomic analysis (Figure 3 and Supplementary Figures 6–9). Volcano plots revealed a few consistent early (3 days) changes in the ileal and colic metabolomes with an Akk-induced increase in N1,N12-diacetylspermine (in both ileum and colon) as well as an increase in colic short-chain fatty acids (day 3: propionate, day 7: butanoic acid, also called butyrate) (Figure 4 and Supplementary Figures 10, 11). A Akk-induced increase in N1-acetylspermidine and N1,N8-diacetylspermidine was observed in the ileum (Supplementary Figure 10) and in the colon on day 7 but not 14 (Supplementary Figure 11). Metabolites generated during the catabolism of branched chain amino acids were also found overabundant in the ileum (day 3: ketoisovaleric acid, ketoisocaproic acid, Figure 4) and the colon (day 7: ketoisocaproic acid, Supplementary Figure 11). Of note, Akk caused the levels of N1,N12-diacetylspermine to increase in the liver at a late time point (day 14, Supplementary Figure 12). Ferulic acid was also increased in Akk-treated mouse livers on days 3 and 14 (Supplementary Figure 12). In contrast, no consistent changes were observed in the plasma after treatment with Akk, perhaps with the exception of a significant but transient (on day 3) increase of 2−hydroxy−3−methylbutyric acid (Supplementary Figure 13). In conclusion, it appears that Akk has the capacity to increase the concentration of N1,N12-diacetylspermine in the gut as well as in the liver.

### Metabolic effects of pasteurized (Akk-past) versus live *Akkermansia muciniphila* (Akk)

**Figure 5:**
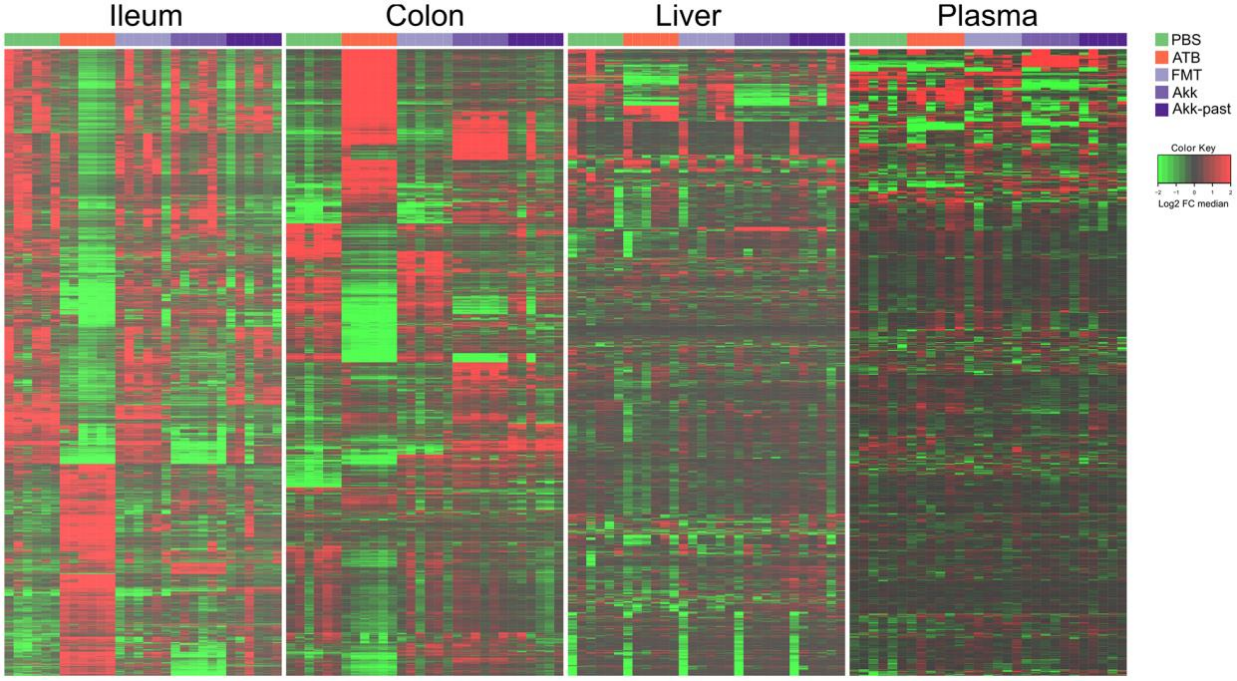
**Metabolic signature obtained through untargeted metabolomic analysis, and illustrated by heatmaps showing the relative abundance changes in metabolites extracted from ileum, colon, liver, and plasma from mice treated with FMT, Akk or with Akk-past (versus PBS or continuous ATB).** Untargeted analysis allowed the identification of more than 24000 and 22000 features in the ileum and the colon samples, respectively, while over 3000 and 1700 features were detected in liver and in plasma samples, respectively. Each row represents a detected feature. Hierarchical clustering (euclidean distance, ward linkage method) of the metabolite abundance is shown. PBS, phosphate buffer saline; ATB, antibiotics; FMT, fecal microbiota transplant; Akk, *Akkermansia muciniphila*; Akk-past, pasteurized *Akkermansia muciniphila*; FC, fold change.

**Figure 6:**
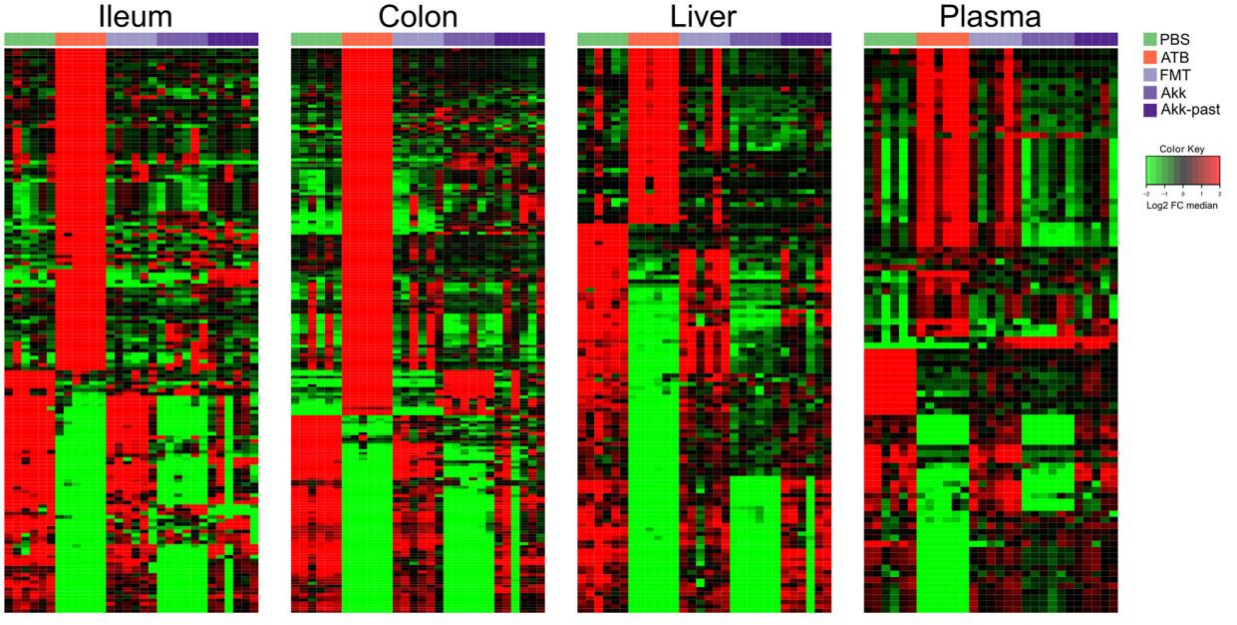
**Reduced picture of the untargeted metabolomics analysis illustrated in Figure 5, showing selected features which significantly increased after Akk or Akk-past oral gavage in the different extracted organs, compared to FMT, PBS, or continuous ATB.** Each row represents a metabolite. Hierarchical clustering (euclidean distance, ward linkage method) of the metabolite abundance is shown. PBS, phosphate buffer saline; ATB, antibiotics; FMT, fecal microbiota transplant; Akk, *Akkermansia muciniphila*; Akk-past, pasteurized *Akkermansia muciniphila*; FC, fold change.

In a subsequent experiment, we specifically addressed the question as to whether Akk can induce long-term changes (day 27) using the aforementioned methodological approach (Figure 1B). We compared the metabolome of control mice (PBS), ATB-treated mice not receiving any bacteria (ATB group), mice receiving FMT alone (FMT group) or mice receiving FMT in combination with live Akk (Akk group) or Akk-past. The overview heatmaps (24000 features in the ileum, 22000 in the colon, 3000 in the liver, 1700 features in plasma) confirm the observations in the first experiment, with major changes in the ileal and colic metabolomes and comparably minor alterations in liver and plasma (Figure 5 and Supplementary Tables 5–8). Plotting the significant changes only (Figure 6), clearly revealed that live Akk has a stronger modulatory effect (as compared to FMT only) than Akk-past.

Nonetheless, as compared to live Akk, Akk-past induced higher levels of several polyamines (N1-acetylspermine, N1,N12-diacetylspermine, N1,N8-diacetylspermidine, spermidine) and their precursor ornithine, and the ornithine/arginine ratio (with no effect on the arginine level), short-chain fatty acids (acetate, propionate), as well as the ketone body 2-hydroxybutyrate (and its likely derivative 2-hydroxy-3-methylbutyrate) in the ileum (Supplementary Figures 14–16 and Supplementary Table 9). Many among these metabolites were also increased with Akk-past, but not with live Akk, in the colon, as observed for several polyamines (N1,N12-diacetylspermine, N1,N8-diacetylspermidine, spermidine, putrescine) an increase of ornithine with a relative depletion of its precursor arginine resulting in an increased ornithine/arginine ratio, as well as for 2-hydroxybutyrate and 2-hydroxy-3-methylbutyrate (Supplementary Figures 14, 15, 17 and Supplementary Table 10). We observed an augmentation of bile acids in response to Akk-past in the ileum (deoxycholic, hyodeoxycholic, murideoxycholic acid) and the colon (deoxycholic, hyocholic, lithocholic, omega−muricholic, murideoxycholic, taurodeoxycholic, tauro−muricholic, taurohyodeoxycholic, tauroursodeoxycholic acids) (Supplementary Figures 16, 17). Outside of the gut, the only consistent increase induced by Akk-past as compared to live Akk also concerned bile acids (such as chenodeoxycholic, deoxycholic, hyodeoxycholic, beta−muricholic, omega−muricholic taurodeoxycholic, ursodeoxycholic acids). This increase in bile acids was only observable in plasma but not in liver (Supplementary Figures 18, 19 and Supplementary Tables 11, 12).

**Figure 7:**
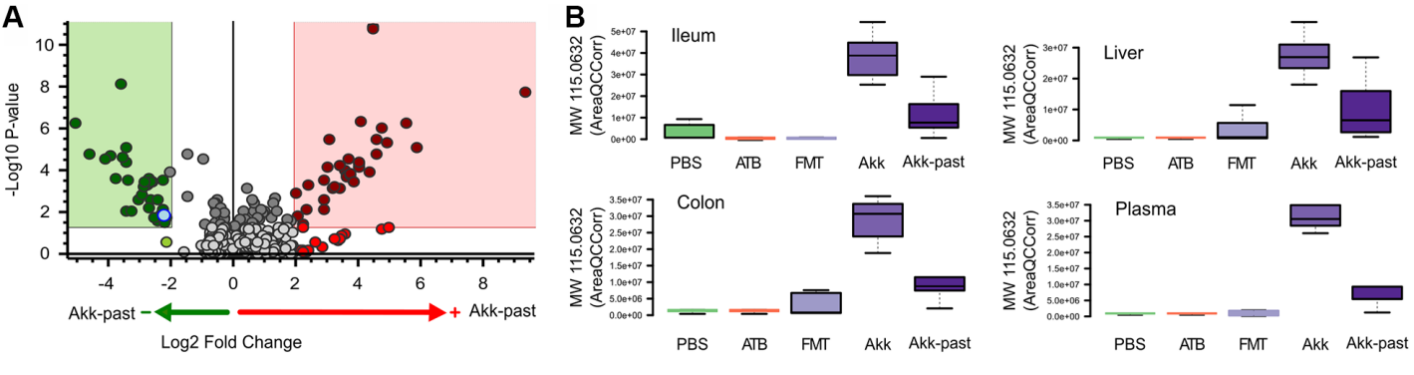
Differential plasma metabolites identification between Akk- or Akk-past-treated mice (**A**). One metabolite (marked in blue in **A**) showed significant difference after comparison versus PBS, ATB and FMT groups, it was annotated by its molecular weight as MW 115.0632. (**B**) Plasma MW 115.0632 was identified in ileum, colon and liver, according to similar retention time and fragments, showing the same trend in the four extracted organs.

In general, targeted analysis showed that Akk live or pasteurized, induced significant changes on the metabolome of the treated mice, being more remarkable in the gut (Supplementary Figure 20). Untargeted analyses of the plasma revealed numerous significant differences in the metabolome of mice receiving FMT plus live Akk versus FMT plus Akk-past (Figure 7A). One metabolite that was selectively induced in ileum, colon, liver and plasma by live Akk, not by Akk-past nor by any other condition, was annotated by its molecular weight (MW 115.0632) (Figure 7B).

In summary, there are important differences in the metabolic effects of live Akk *versus* Akk-past that may be biologically relevant because they affect multiple bile acids, a ketone body, several polyamines, as well as short chain fatty acids.

## DISCUSSION

In this work, we report that Akk, especially if it is pasteurized, causes major changes in metabolism, elevating the concentrations of several metabolites that have been previously associated with positive effects on health. These results strongly suggest that Akk does not act in a direct fashion but rather impacts the gut microbiota through an indirect mode of action, by creating the conditions to produce “good metabolites” and “good bacteria”.

Indeed, Akk expresses a heat-resistant surface protein, Amuc_1100, which acts on Toll-like receptors 2 and 4 expressed by host cells [18, 27]. Recombinant Amuc_1100 protein ameliorates transepithelial electrical resistance in Caco-2 monolayers *in vitro*, suggesting that it can improve the gut barrier function in a direct fashion [27]. Moreover, such direct effects of Amuc_1100 might account for the Akk-induced stimulation of mucin production by enterocytes and then modify the intestinal microbiota, causing indirect effects. However, a study with overweight and obese humans revealed that supplementation with either live Akk or Akk-past did not affect the overall structure of the gut microbiome [19]. In line with this finding, live Akk supplemented to mice did not significantly affect the gut microbiota [28], arguing against such indirect effects mediated by other bacteria. Since it has been shown that Akk-past is able to reduce body-weight and fat mass gain through mechanisms implying a reduction on carbohydrate absorption, higher intestinal epithelial cell turnover, and increased excretion of energy in the feces [29], it is reasonable to suggest that the observed changes in the metabolome are related to an Amuc_1100-induced metabolic effects rather than major alterations in the gut microbiota.

The gene coding for Amuc_1100 can be transferred into *Escherichia coli*, which then acquires Akk-like features with respect to the suppression of obesity [30]. Moreover, recombinant Amuc_1100 has antiobesity [18] as well as anti-inflammatory effects in mice, for example in mouse models of colitis [31] and experimental periodontitis [32]. A clinical study that compared the effects of pasteurized versus live Akk led to the conclusion that the pasteurized bacterium was more efficient than the live one [19]. This appears counterintuitive but may reflect the importance of the direct effects of the bacterium (and Amuc_1100) on intestinal metabolism. Speculatively, it is possible that pasteurization increases the bioavailability of Amuc_1100 by effects on bacterial surface charges or the structure of the membrane. Alternatively, pasteurization of Akk could prevent the production of metabolites or factors that mitigate its beneficial effects.

Akk-past was more efficient than live Akk in elevating the intestinal concentrations of polyamines, short-chain fatty acids, 2-hydroxybutyrate, as well multiple bile acids, which also increased in the circulation. All these metabolites may have a positive impact on human health.

Polyamines such as spermidine and spermine, as well as their acetylated metabolites are known for their antiaging effects across phylogeny (in yeast, nematodes, flies and mice) [26, 33–35]. The nutritional uptake of polyamines has been correlated with reduced mortality from cancer and cardiovascular disease in two independent population studies [36, 37]. Moreover, in mice, spermidine improve the gut barrier function [38] and has marked antiobesity, antidiabetic and cancer immunosurveillance-improving effects [39–41]. These effects of polyamines are due to the induction of autophagy in host cells [40, 42, 43]. Hence, it will be important to investigate whether Akk induces autophagy in the gut or other organs including the liver [44]. Moreover, it will be interesting to test the possibility to further increase polyamine synthesis by oral supplementation of arginine, together with Akk, as this has been documented in a prior publication [45].

Short-chain fatty acids (such as butyrate and propionate), which are generated from fermentable fibers, contribute to the maintenance of the gut barrier function through a contribution to energy metabolism of enterocytes as well as an action on the metabolite-sensing G protein-coupled receptors GPR41, GPR43 and GPR109A by gut epithelia and immune cells [46, 47]. Of note, short-chain fatty acid-producing taxa (such as Ruminococcaceae, Faecalibacterium) are associated with health [48]. Moreover, the plasma concentrations of butyrate and propionate are associated with protection from chronic graft-versus-host disease in patients [49], as well as with the therapeutic response of cancer patients to PD-1 blockade [50, 51].

The ketone body 2-hydroxybutyrate can be administered to mice and stimulates anticancer immune responses in the context of immune checkpoint [52]. 2-hydroxybutyrate is increased in the context of ketogenic diets, which are known for their antiaging effects [53–55], as well as their capacity to stimulate anticancer immunity [56]. Finally, an increase in bile acids has been associated with the antiaging effects of methionine restriction and FMT in a mouse model [17, 57].

In summary, Akk has pleiotropic metabolic effects that may explain its capacity to support gut homeostasis and organismal health. It appears plausible that the simultaneous elevation of multiple metabolites with known antiaging and anticancer effects account for the bodywide action of Akk.

## MATERIALS AND METHODS

All antibiotics, standards, solvents, and reagents were bought from Sigma Aldrich, unless stated otherwise.

### Mice

Experiments were performed in accordance with Government and institutional guidelines and regulations (French and European laws and regulations). The institutional board of animal ethics and Ministère de la Recherche approved all mouse experimental settings (permission numbers: 2014-074-501 and APAFIS#13366-2018020510263031v3). Female C57BL/6 were purchased and used at 7 weeks of age from Envigo (France) and bred in specific pathogen-free conditions (unless otherwise specified) in the animal facility of Gustave Roussy Cancer Campus.

### Antibiotic treatments

Intestine decontamination was achieved with an antibiotic solution (ATB) containing ampicillin (1 mg/mL), streptomycin (5 mg/mL) and colistin (1 mg/mL), added to the sterile drinking water of mice. Duration of ATB treatment was different based on the experimental settings (Figure 1A, 1B). Antibiotic activity was confirmed by cultivating fecal pellets resuspended in Brain Heart Infusion (BHI) + 15% glycerol at 0.1 g/mL on COS (Columbia Agar with 5% Sheep Blood, COS ref 43049, BioMérieux, France) plates for 48h at 37° C in aerobic and anaerobic conditions (GENbox Jar and GENbag, Biomérieux, France).

### Fecal microbiota transplantation (FMT) experiments

Mice received 3 days of ATB before undergoing FMT the next day by oral gavage using animal feeding needles. FMT was performed by thawing fecal material. Two hundred μL of the suspension was then transferred by oral gavage into ATB pre-treated recipients. In addition, another 100μL was applied on the fur of each animal.

### Bacterial preparations and gut colonization with dedicated bacterial species

Colonization of ATBs pre-treated or FMT-reconstituted C57BL/6 mice was performed by oral gavage with 100 μL of suspension containing 1×10^8^ bacteria. *Enterococcus hirae* were grown on COS plates for 24 h at 37° C in aerobic conditions while other bacteria, including *A. muciniphila* p2261, were grown on COS plates for 72 h at 37° C in anaerobic conditions. In some experiments, an identical quantity of grown *A. muciniphila* was inactivated by pasteurization for 30 min at 70° C, and then immediately frozen and stored at −80° C [19]. Colonization of 3-day ATB pre-treated or FMT-treated C57BL/6 mice was performed by oral gavage with 100 μL of suspension containing between 1×10^8^ and 1×10^9^ bacteria. Culture and identification of specific bacteria was accomplished through the combination of morphological tests and analysis by means of a Matrix-Assisted Laser Desorption/ Ionisation Time of Flight (MALDI-TOF) mass spectrometer (Andromas, Beckman Coulter, France or Microflex, Bruker). Five bacterial gavages were performed for each mouse, the first 24 h after stopping ATB administration. We next monitored in a kinetic manner (day 3, day 7, day 14) the impact of oral gavages with several commensals on the local (ileal, colonic) and distal (liver, plasma) concentrations of various metabolites using gas and liquid chromatography coupled to mass spectrometry as described below.

### Sample preparation tissue (liver) and fecal content (ileum, colon)

About 30 mg of biological matrix for each condition were first weighted and solubilized into 1.5 mL polypropylene microtubes with ceramic beads with 1 mL of cold lysate buffer with internal standard (ISTD) (methanol/water/chloroform, 9/1/1, -20° C). They were then homogenized three times for 20 s at 5500 rpm using Precellys 24 tissue homogenizer (Bertin Technologies, Montigny-le-Bretonneux, France), followed by a centrifugation (10 min at 15000 g, 4° C). Then the upper phase of supernatant was split in three parts: first 300 μL were used for Gas Chromatography coupled to Mass Spectrometry (GC/MS) experiment in microtube. Next, two aliquots (50 μL and 300 μL, respectively) were used for Ultra High Pressure Liquid Chromatography coupled to Mass Spectrometry (UHPLC/MS) analysis. Concerning GC-MS aliquots, 300 μL were transferred from the microtube to glass tube and evaporated. 50 μL of methoxyamine (20 mg/mL in pyridine) were added on dried extracts, then stored at room temperature in dark, during 16 hours. The day after, 80 μL of MSTFA were added and final derivatization occurred at 40° C during 30 min. Samples were then transferred in vials and directly injected into GC-MS. Concerning UHPLC-MS aliquots (for short chain fatty acids, SCFA method), 50 μl were transferred from the injection vial, and mixed with 25μl of 3-Nitrophenylhydrazine (3-NPH, 200 mM) and 25 μl of *N*-(3-Dimethylaminopropyl)-*N*′-ethylcarbodiimide hydrochloride (EDC, 150 mM). The whole was heated at 40° C during 1h. Next, 100 μl of water were added and samples were injected into UHPLC-MS. Concerning the other UHPLC-MS aliquots, 300 μL were evaporated in microtubes at 40° C in a pneumatically-assisted concentrator (Techne DB3*, Staffordshire, UK*). UHPLC-MS dried extracts were solubilized with 200 μL of MilliQ water. Aliquots for analysis were transferred in LC vials and injected into UHPLC-MS or kept at -80° C until injection. Regarding the rest of the supernatant and the pellet, 200 μl of methanol with 2% of sulfosalicylic acid (SSA) were added before vortex and centrifugation (10 min at 15000 g, 4° C). Next, 200 μl of the supernatant were transferred in a microtube and evaporated. The dried samples were spiked with 200 μl of MilliQ water transferred in polypropylene vials before injection in UHPLC/MS for polyamines analysis.

### Sample preparation plasma (lithium heparin)

A volume of 25 μL of plasma was mixed with 250 μL of cold solvent mixture with ISTD (methanol/water/chloroform, 9/1/1, -20° C), into 1.5 mL microtube, vortexed and centrifugated (10 min at 15000 g, 4° C). Then the sample preparation followed the same step as for the tissues and fecal contents: 50 μL were used for GC-MS experiment in glass tubes, 50 μL for UHPLC-MS analysis, and 40 μL were used for short chain fatty acids detection. Regarding the rest of the supernatant and the pellet, 85 μl of methanol with 2% of sulfosalicylic acid (SSA) were added before vortex and centrifugation (10 min at 15000g, 4° C). 200 μl of the supernatant were transferred into a microtube and evaporated. The dried samples were spiked with 200 μl of MilliQ water transferred in polypropylene vials before injection in UHPLC/MS for polyamines analysis.

### Quality control policy

A daily qualification of the instrumentation was set up with automatic tune and calibration processes. These qualifications were completed with double injections of standard mix, at the beginning and end of the run, as for a blank extracted sample to control background impurities. Standard mixes were adapted for each chromatographic method. After extraction, fractions of each biological sample were pooled to create a quality control (QC) sample, use to passivate the column before analysis with proper biological matrix and re-injected throughout the batch to monitor and correct analytical bias occurring during batch (*m*/*z*, retention time and sensitivity drifts) during post acquisition treatment signal.

### Widely-targeted analysis of intracellular metabolites by gas chromatography (GC) coupled to a triple quadrupole (QQQ) mass spectrometer

GC-MS/MS method was performed on a 7890A gas chromatography (Agilent Technologies, Santa Clara, CA, USA) coupled to a triple quadrupole 7000C (Agilent Technologies,) equipped with a high sensitivity electronic impact source (EI) operating in positive mode. Detailed analytical methods are described in [26].

### Targeted analysis of bile acids by ion pairing ultra-high performance liquid chromatography (UHPLC) coupled to a triple quadrupole (QQQ) mass spectrometer

Targeted analysis was performed on a RRLC 1260 system (Agilent Technologies) coupled to a Triple Quadrupole 6410 (Agilent Technologies) equipped with an electrospray source operating in negative mode. Gas temperature was set to 325° C with a gas flow of 12 L/min. Capillary voltage was set to 4.5 kV [26]. Peak detection and integration of analytes were performed using the Agilent Mass Hunter quantitative software (B.07.01), exported as tables and processed within R software.

### Targeted analysis of polyamines by ion pairing ultra-high performance liquid chromatography (UHPLC) coupled to a triple quadrupole (QQQ) mass spectrometer

Targeted analysis was performed on a RRLC 1260 system (Agilent Technologies) coupled to a Triple Quadrupole 6410 (Agilent Technologies) equipped with an electrospray source operating in positive mode. The gas temperature was set to 350° C with a gas flow of 12 l/min. The capillary voltage was set to 3.5 kV [26]. Peak detection and integration of analytes were performed using the Agilent Mass Hunter quantitative software (B.07.01), exported as tables and processed within R software.

### Targeted analysis of short chain fatty acids by ion pairing ultra-high performance liquid chromatography (UHPLC) coupled to a triple quadrupole (QQQ) mass spectrometer

Targeted analysis was performed on a RRLC 1260 system (Agilent Technologies) coupled to a Triple Quadrupole 6410 (Agilent Technologies) equipped with an electrospray source operating in negative mode. Gas temperature was set to 350° C with a gas flow of 12 L/min. Capillary voltage was set to 4.0 kV [26]. Peak detection and integration of analytes were performed using the Agilent Mass Hunter quantitative software (B.07.01), exported as tables and processed within R software.

### Pseudo-targeted analysis of intracellular metabolites by ultra-high performance liquid chromatography (UHPLC) coupled to a Q-Exactive mass spectrometer. Reversed phase acetonitrile method

The profiling experiment was performed with a Dionex Ultimate 3000 UHPLC system (Thermo Scientific) coupled to a Q-Exactive (Thermo Scientific, Waltham, MA, USA) equipped with an electrospray source operating in both positive and negative mode and full scan mode from 100 to 1200 m/z. The Q-Exactive parameters were: sheath gas flow rate 55 au, auxiliary gas flow rate 15 au, spray voltage 3.3 kV, capillary temperature 300° C, S-Lens RF level 55 V. The mass spectrometer was calibrated with sodium acetate solution dedicated to low mass calibration. 10 μL of sample were injected on a SB-Aq column (100 mm × 2.1 mm particle size 1.8 μm, Agilent Technologies), protected by a guard column XDB-C18 (5 mm × 2.1 mm particle size 1.8 μm) and heated at 40° C by a pelletier oven. The gradient mobile phase consists of water with 0.2% of acetic acid (A) and acetonitrile (B). The flow rate was set to 0.3 mL/min. Initial condition is 98% phase A and 2% phase B. Molecules were then eluted using a gradient from 2% to 95% phase B in 22 min. The column was washed using 95% mobile phase B for 2 minutes and equilibrated using 2% mobile phase B for 4 min. The autosampler was kept at 4° C. Peak detection and integration were performed using the Thermo Xcalibur quantitative software (version 2.2).

### Untargeted analysis of intracellular metabolites by ultra-high performance liquid chromatography (UHPLC) coupled to a Q-Exactive mass spectrometer. Reversed phase acetonitrile method

Orbitrap mass spectrometer (q-Exactive, Thermo Scientific) operating in both negative and positive ion, was used coupled to an UHPLC system (Ultimate 3000 UHPLC, Dionex) and acquired samples in full scan analysis mode. LC separation was performed on reversed phase (Zorbax Sb-Aq 100 x 2.1mm x 1.8μm, Agilent; A: water 0.2% acetic acid, B: ACN). The details about the analytical method are described above. Data acquisition was performed with the Thermo Scientific Xcalibur software (version 2.2). Raw data files were used to perform unbiased profiling analysis, with Thermo Scientific Compound Discoverer small molecule identification software (version 3.1).

### Data analysis using compound discoverer

Raw data files obtained by the previously described pseudo-targeted analysis were also used to perform unbiased profiling analysis, using the Thermo Compound Discoverer (3.1.). After sample injection and data acquisition, raw data files were processed with Compound Discoverer software following a customized node-based workflow for identifying unknown compounds in metabolomics. First, spectra selection and retention time alignment were performed, followed by removal of background noise and baseline correction. Next, the processing workflow found chromatographic peaks for unknown compounds (molecular weight, MW, x retention time, RT) extracting all relevant spectral and chromatographic information, to predict the elemental composition of the unknowns. The possible identity of the unknown compounds was then searched against selected MS databases, such as ChemSpider (from MS1 scans by using MW or predicted composition when available), mZcloud (MS/MS spectral library), built-in databases (custom, local libraries), and Metabolika or KEGG databases (metabolic pathway search). Annotations are assigned to the detected compounds, to rank putative database results. Finally, the software performed statistical analysis using a multivariate method approach, e. g. PCA (unsupervised), and data visualization, e.g. volcano plots.

### Statistical analysis

All targeted and pseudo-targeted treated data were merged and cleaned with a dedicated R (version 3.4) package (@Github/Kroemerlab/GRMeta). Calculations and statistical tests were performed using R v3.4. Wilcoxon-Mann-Whitney test was used to assess differences in concentration between two different groups. Data representation was performed with softwares R v3.6 and Rstudio v1.2.1335 using tidyverse, dplyr, ggplot2, ggpubr, complexheatmap and corrplot packages.

## Supplementary Material

Supplementary Figures

Supplementary Table 1

Supplementary Table 2

Supplementary Table 3

Supplementary Table 4

Supplementary Table 5

Supplementary Table 6

Supplementary Table 7

Supplementary Table 8

Supplementary Table 9

Supplementary Table 10

Supplementary Table 11

Supplementary Table 12
