# Supplementary material for: Oral administration of Akkermansia muciniphila elevates systemic antiaging and anticancer metabolites
Source: Aging (Albany NY). 2021 Mar 2;13(5):6375–405. doi: 10.18632/aging.202739 (PMC7993698; doi:10.18632/aging.202739)
Supplement: Supplementary Figures [file aging-13-202739-s001.pdf]

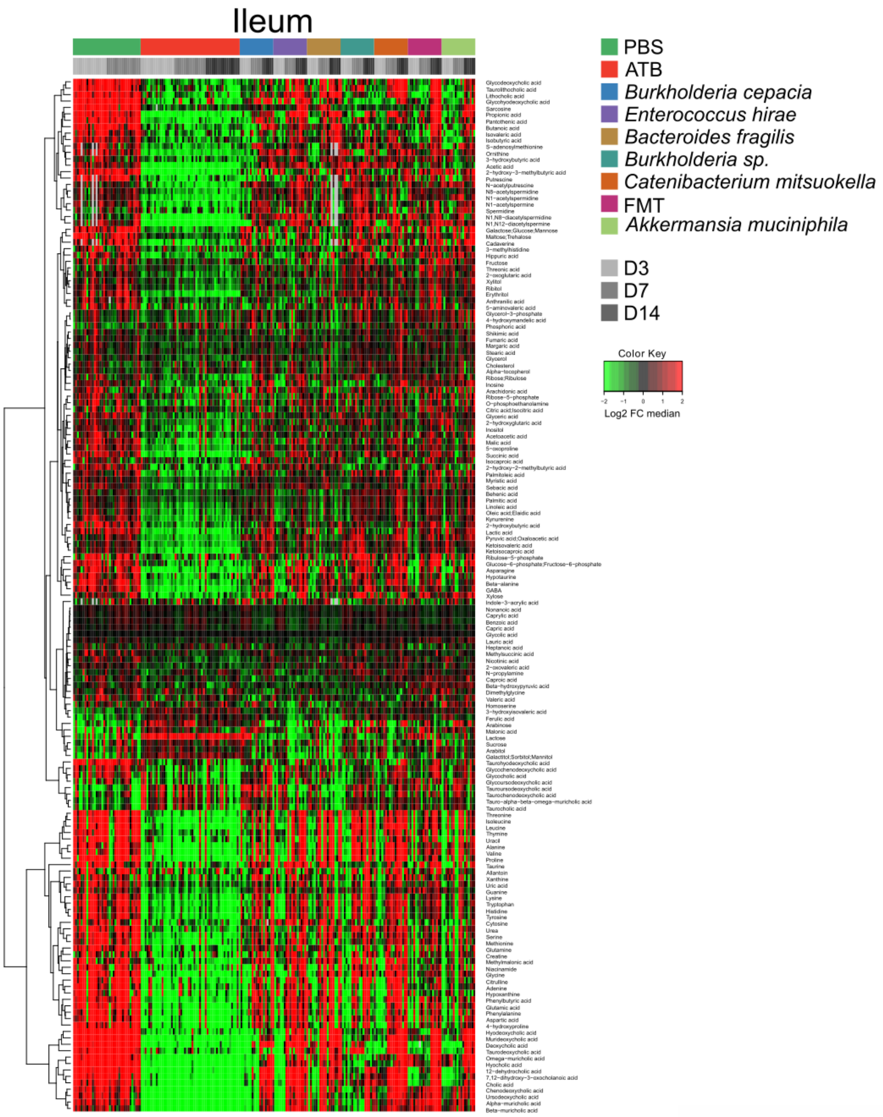

Supplementary Figure 1. Targeted metabolomics analysis was performed on ileum samples from mice receiving oral gavages with several commensals, PBS, or continuous ATB, at days 3, 7 and 14 after the first oral gavage. Changes in metabolites relative abundance are illustrated. Metabolites names and clustering are showed. Hierarchical clustering (euclidean distance, ward linkage method) of the metabolite abundance is shown. ATB, antibiotics; PBS, phosphate buffer saline; FMT, fecal microbiota transplant; FC, fold change.

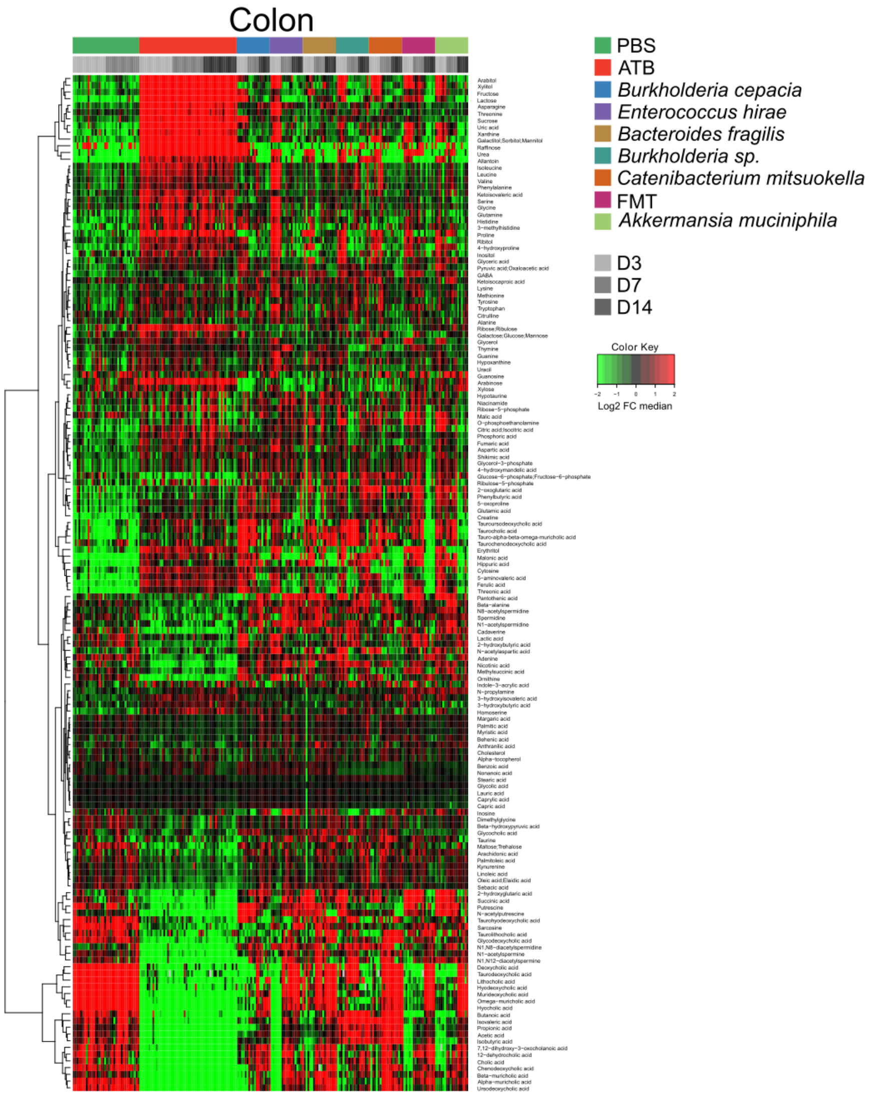

Supplementary Figure 2. Targeted metabolomics analysis was performed on colon samples from mice receiving oral gavages with several commensals, PBS, or continuous ATB, at days 3, 7 and 14 after the first oral gavage. Changes in metabolites relative abundance are illustrated. Metabolites names and clustering are showed. Hierarchical clustering (euclidean distance, ward linkage method) of the metabolite abundance is shown. ATB, antibiotics; PBS, phosphate buffer saline; FMT, fecal microbiota transplant; FC, fold change.

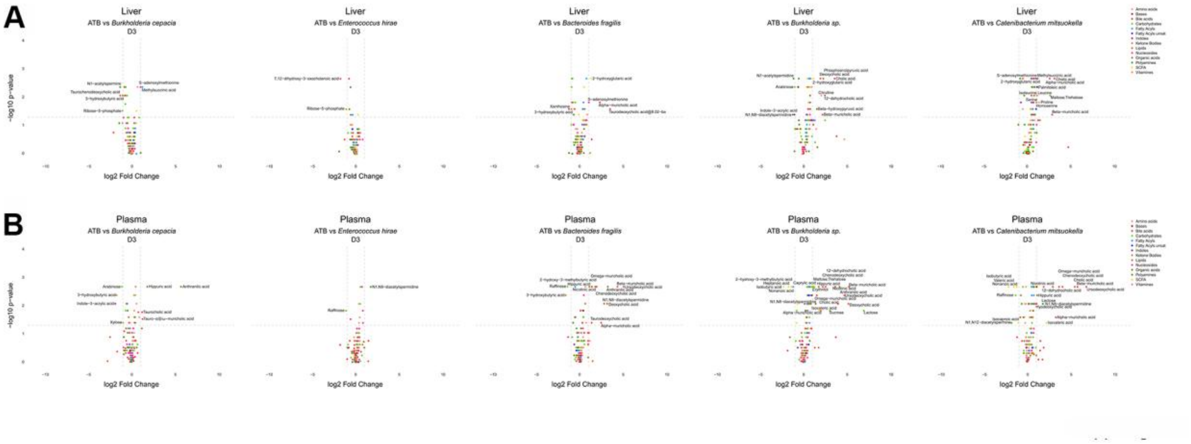

Supplementary Figure 3. Differential metabolite identification in liver (**A**) and in plasma (**B**) samples from mice receiving oral gavages with several commensals *versus* continuous ATB at day 3 after the first oral gavage. The horizontal dashed gray line shows where p=0.05 with points above being metabolites with significantly different relative abundance (p<0.05). The vertical dashed gray lines correspond to FC=1. Targeted metabolites that display both large magnitude FC and higher statistical difference (-log10 of p value) are annotated. Families of metabolites are grouped by colors.

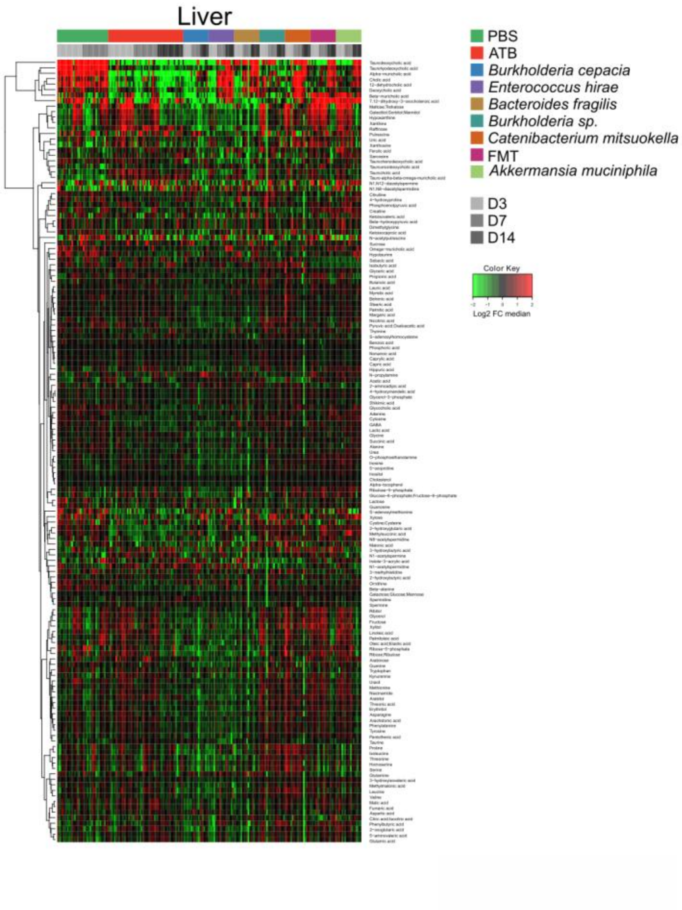

Supplementary Figure 4. Targeted metabolomics analysis was performed on liver samples from mice receiving oral gavages with several commensals, PBS, or continuous ATB, at days 3, 7 and 14 after the first oral gavage. Changes in metabolites relative abundance are illustrated. Metabolites names and clustering are showed. Hierarchical clustering (euclidean distance, ward linkage method) of the metabolite abundance is shown. ATB, antibiotics; PBS, phosphate buffer saline; FMT, fecal microbiota transplant; FC, fold change.

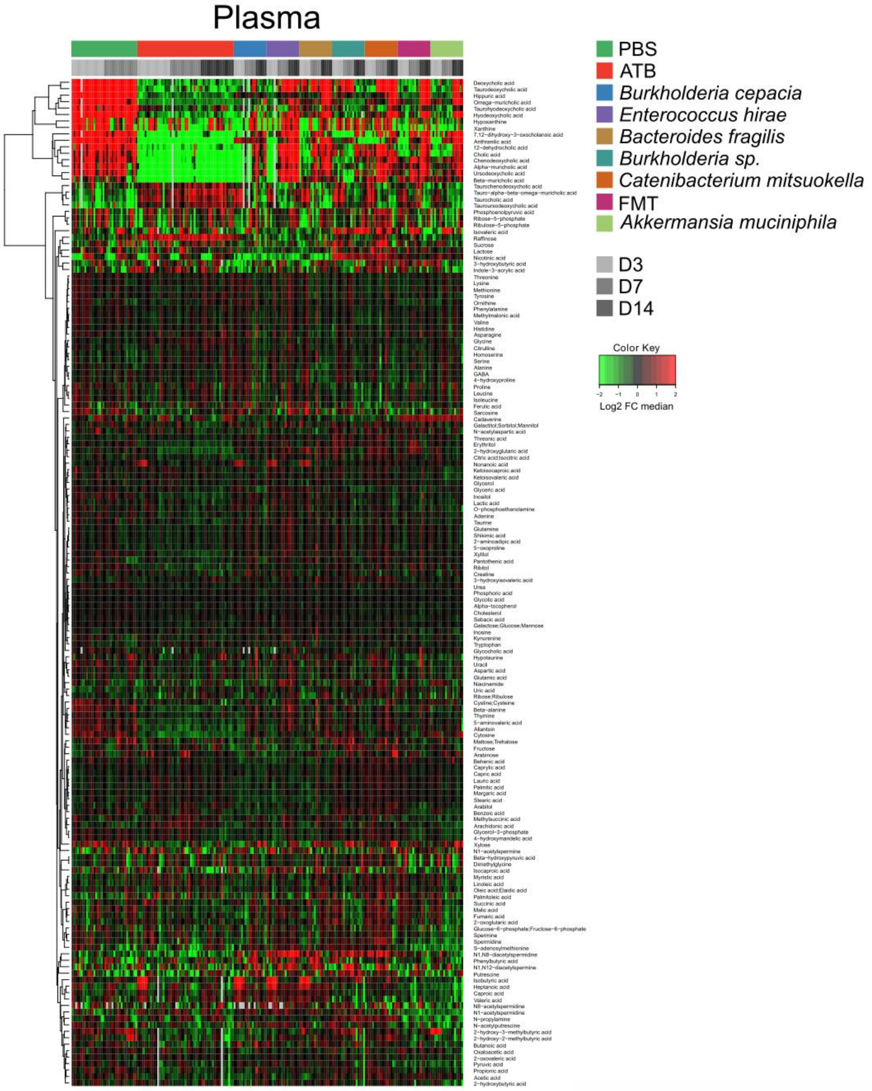

Supplementary Figure 5. Targeted metabolomics analysis was performed on plasma samples from mice receiving oral gavages with several commensals, PBS, or continuous ATB, at days 3, 7 and 14 after the first oral gavage. Changes in metabolites relative abundance are illustrated. Metabolites names and clustering are showed. Hierarchical clustering (euclidean distance, ward linkage method) of the metabolite abundance is shown. ATB, antibiotics; PBS, phosphate buffer saline; FMT, fecal microbiota transplant; FC, fold change.

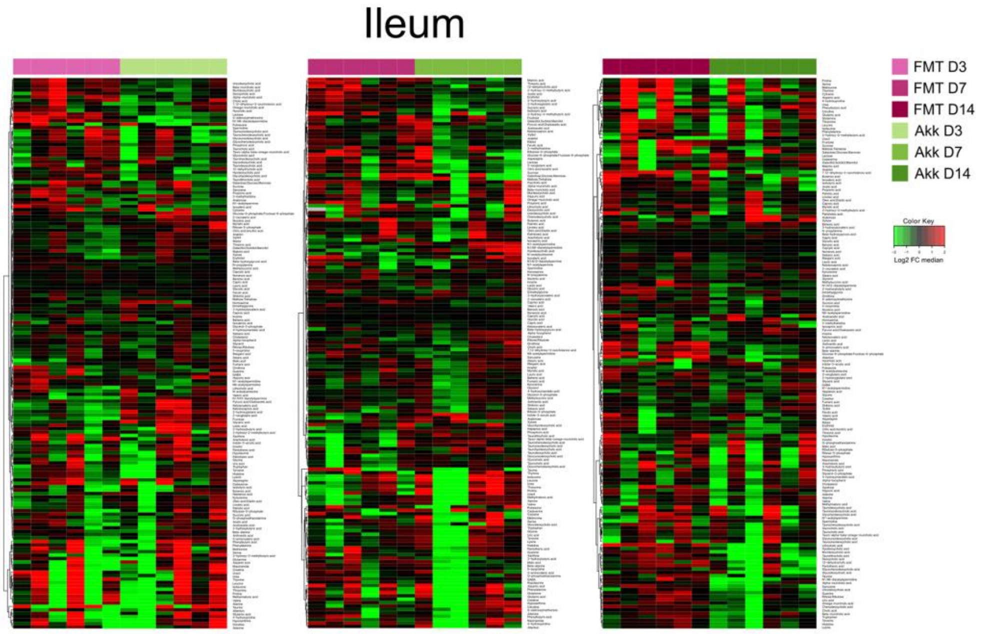

Supplementary Figure 6. Ileum targeted metabolites relative abundance variations in mice treated with FMT or Akk at days 3, 7 and 14 after the first oral gavage. Metabolites names and clustering are showed. Hierarchical clustering (euclidean distance, ward linkage method) of the metabolite abundance is shown. FMT, fecal microbiota transplant; Akk, *Akkermansia muciniphila*; FC, fold change.

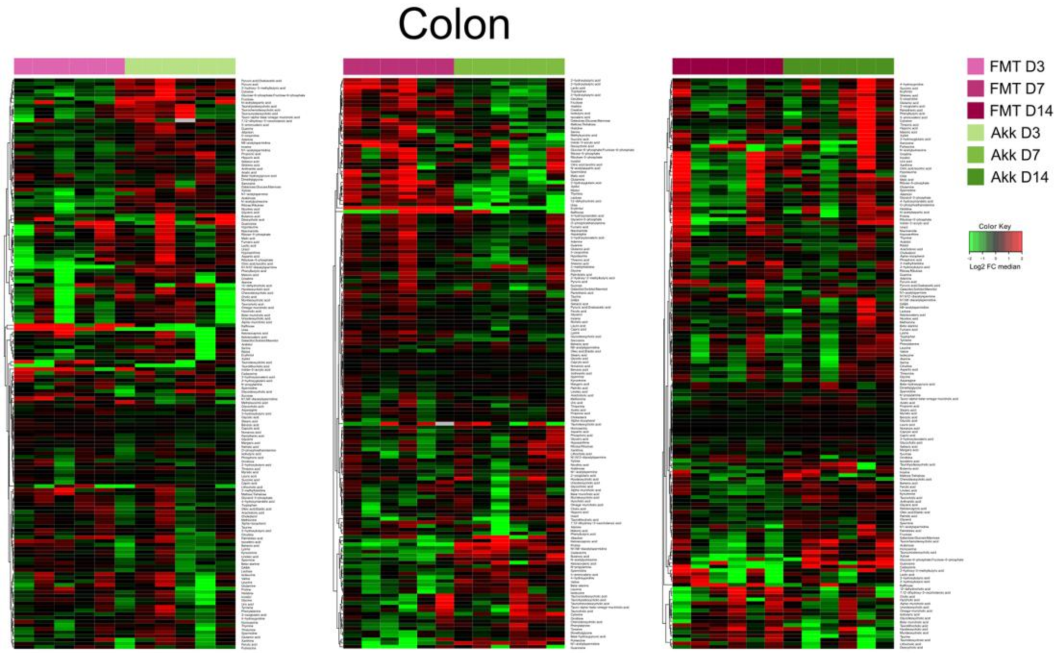

Supplementary Figure 7. Colon targeted metabolites relative abundance variations in mice treated with FMT or Akk at days 3, 7 and 14 after the first oral gavage. Metabolites names and clustering are showed. Hierarchical clustering (euclidean distance, ward linkage method) of the metabolite abundance is shown. FMT, fecal microbiota transplant; Akk, *Akkermansia muciniphila*; FC, fold change.

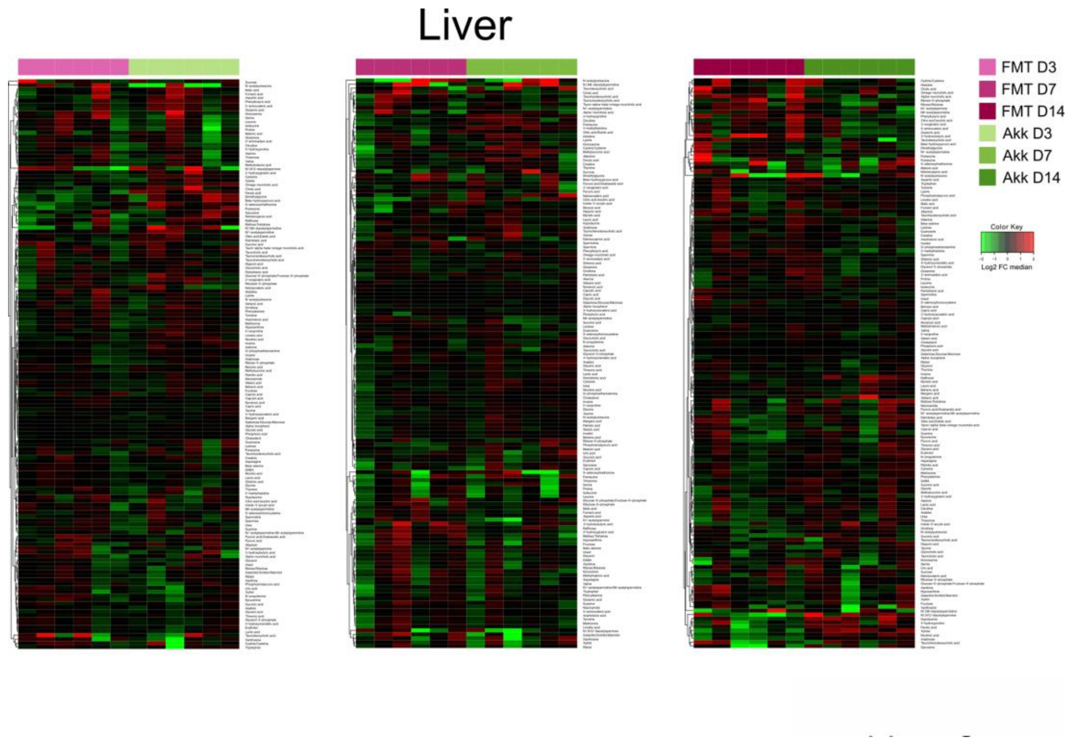

Supplementary Figure 8. Liver targeted metabolites relative abundance variations in mice treated with FMT or Akk at days 3, 7 and 14 after the first oral gavage. Metabolites names and clustering are showed. Hierarchical clustering (euclidean distance, ward linkage method) of the metabolite abundance is shown. FMT, fecal microbiota transplant; Akk, *Akkermansia muciniphila*; FC, fold change.

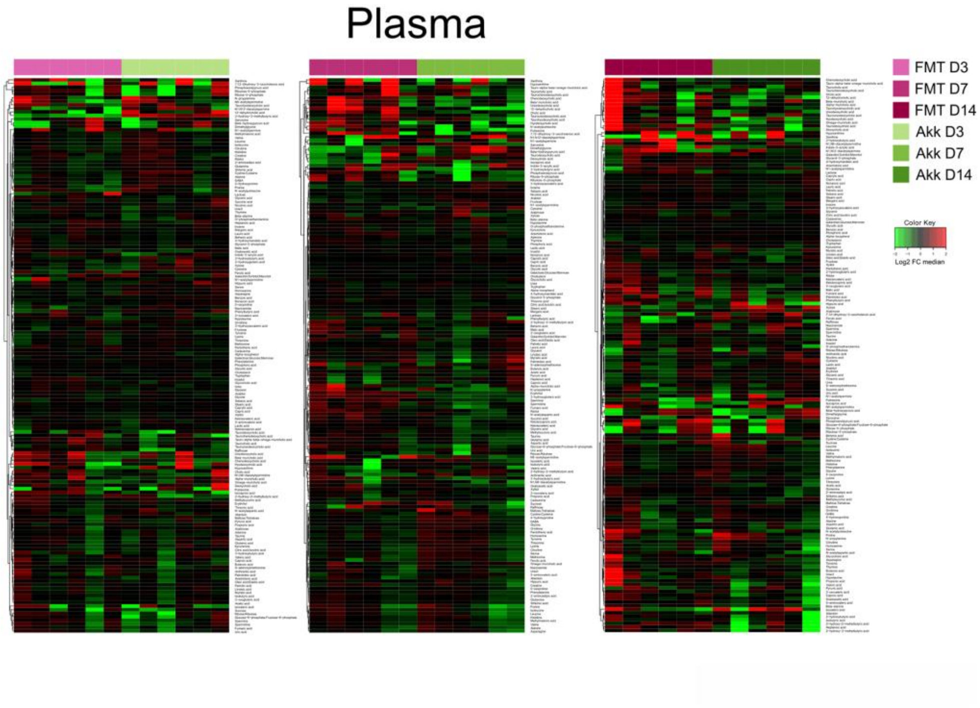

Supplementary Figure 9. Plasma targeted metabolites relative abundance variations in mice treated with FMT or Akk at days 3, 7 and 14 after the first oral gavage. Metabolites names and clustering are showed. Hierarchical clustering (euclidean distance, ward linkage method) of the metabolite abundance is shown. FMT, fecal microbiota transplant; Akk, *Akkermansia muciniphila*; FC, fold change.

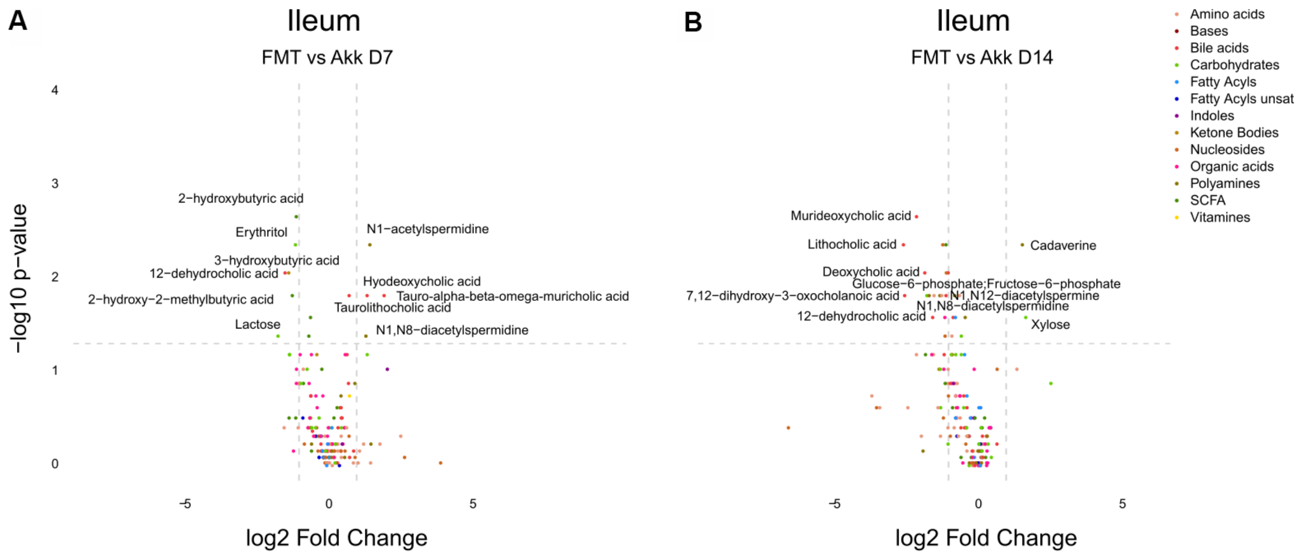

Supplementary Figure 10. Differential metabolite identification in ileum samples from mice receiving FMT or Akk at day 7 (**A**), or at day 14 (**B**) after the first oral gavage. The horizontal dashed gray line shows where *p*=0.05 with points above being metabolites with significantly different relative abundance (*p*<0.05). The vertical dashed gray lines correspond to FC=1. Targeted metabolites that display both large magnitude FC and higher statistical difference (-log10 of *p* value) are annotated. Families of metabolites are grouped by colors.

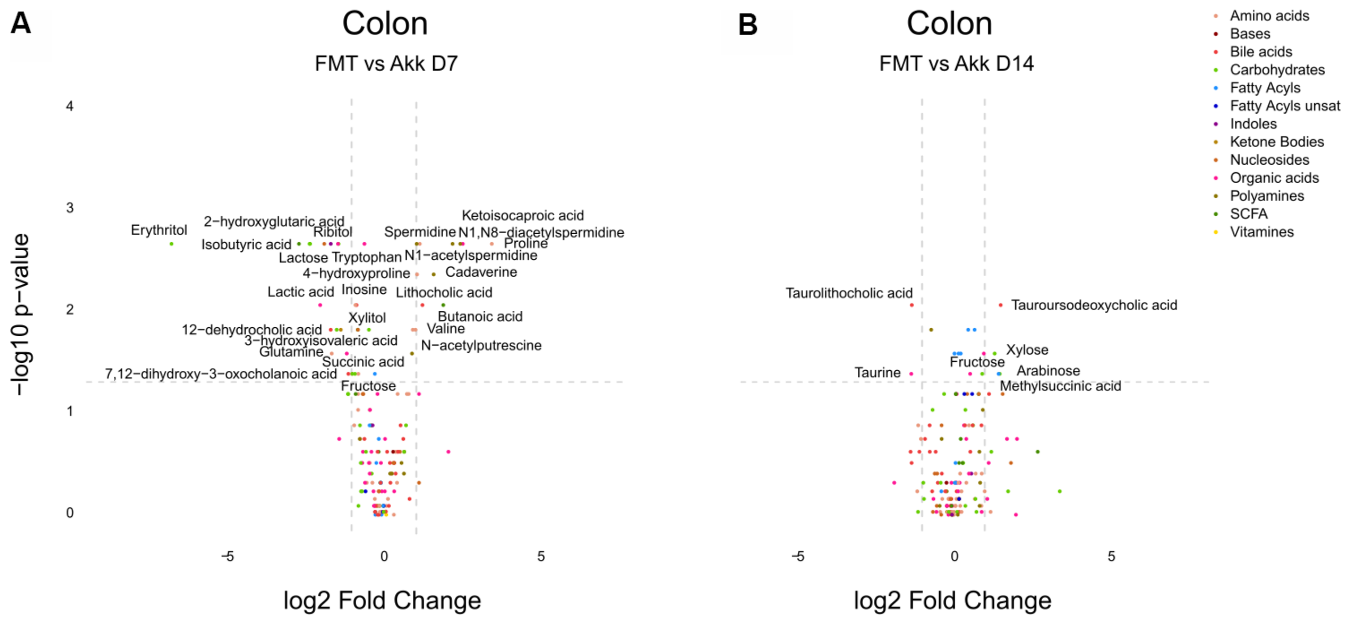

Supplementary Figure 11. Differential metabolite identification in colon samples from mice receiving FMT or Akk at day 7 (**A**), or at day 14 (**B**) after the first oral gavage. The horizontal dashed gray line shows where *p*=0.05 with points above being metabolites with significantly different relative abundance (*p*<0.05). The vertical dashed gray lines correspond to FC=1. Targeted metabolites that display both large magnitude FC and higher statistical difference (-log10 of *p* value) are annotated. Families of metabolites are grouped by colors.

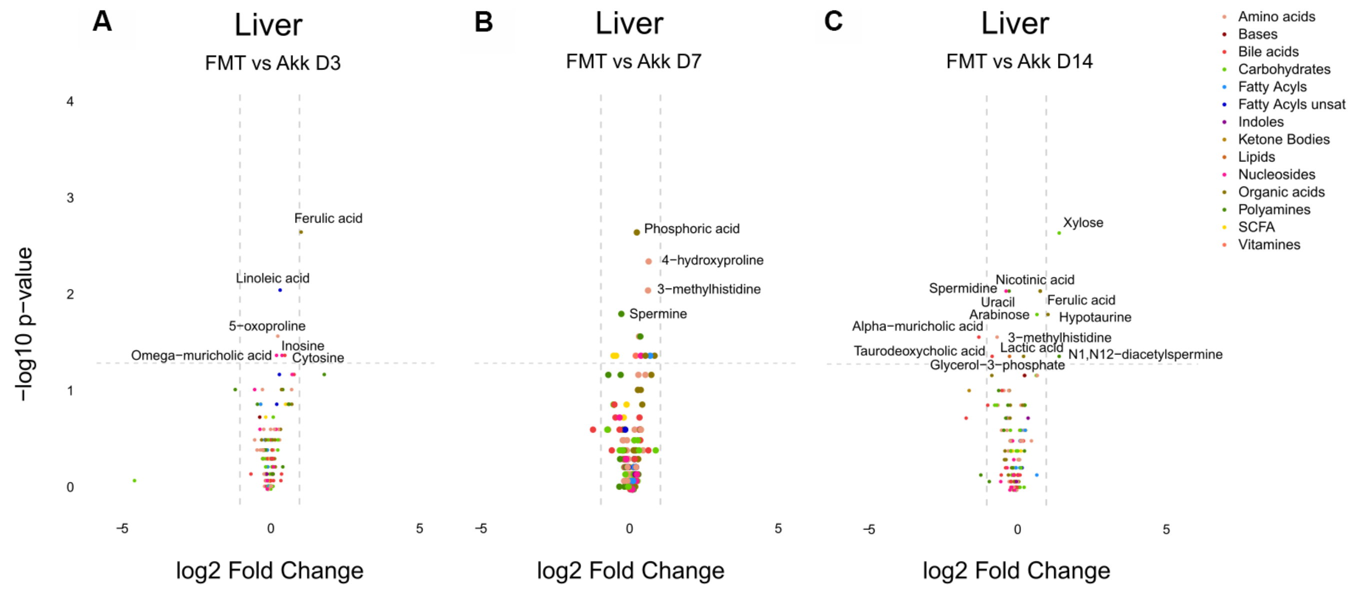

Supplementary Figure 12. Differential metabolite identification in liver samples from mice receiving FMT or Akk at day 3 (**A**), day 7 (**B**) or at day 14 (**C**) after the first oral gavage. The horizontal dashed gray line shows where *p*=0.05 with points above being metabolites with significantly different relative abundance (*p*<0.05). The vertical dashed gray lines correspond to FC=1. Targeted metabolites that display both large magnitude FC and higher statistical difference (-log10 of *p* value) are annotated. Families of metabolites are grouped by colors.

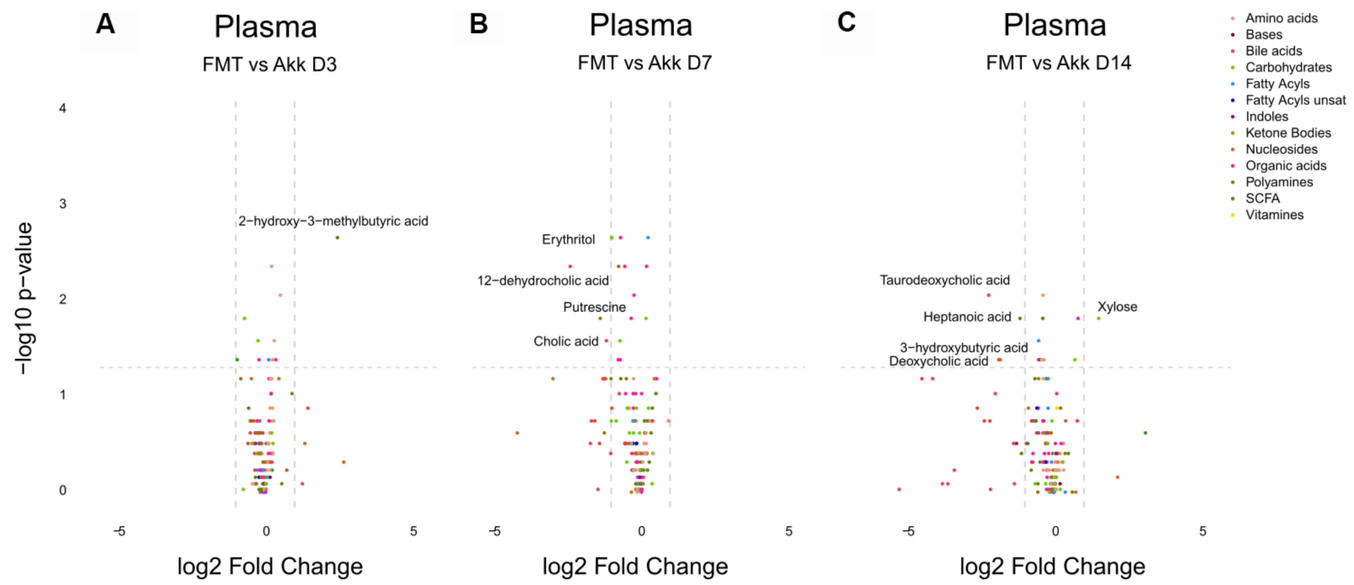

Supplementary Figure 13. Differential metabolite identification in plasma samples from mice receiving FMT or Akk at day 3 (**A**), day 7 (**B**) or at day 14 (**C**) after the first oral gavage. The horizontal dashed gray line shows where *p*=0.05 with points above being metabolites with significantly different relative abundance (*p*<0.05). The vertical dashed gray lines correspond to FC=1. Targeted metabolites that display both large magnitude FC and higher statistical difference (-log10 of *p* value) are annotated. Families of metabolites are grouped by colors.

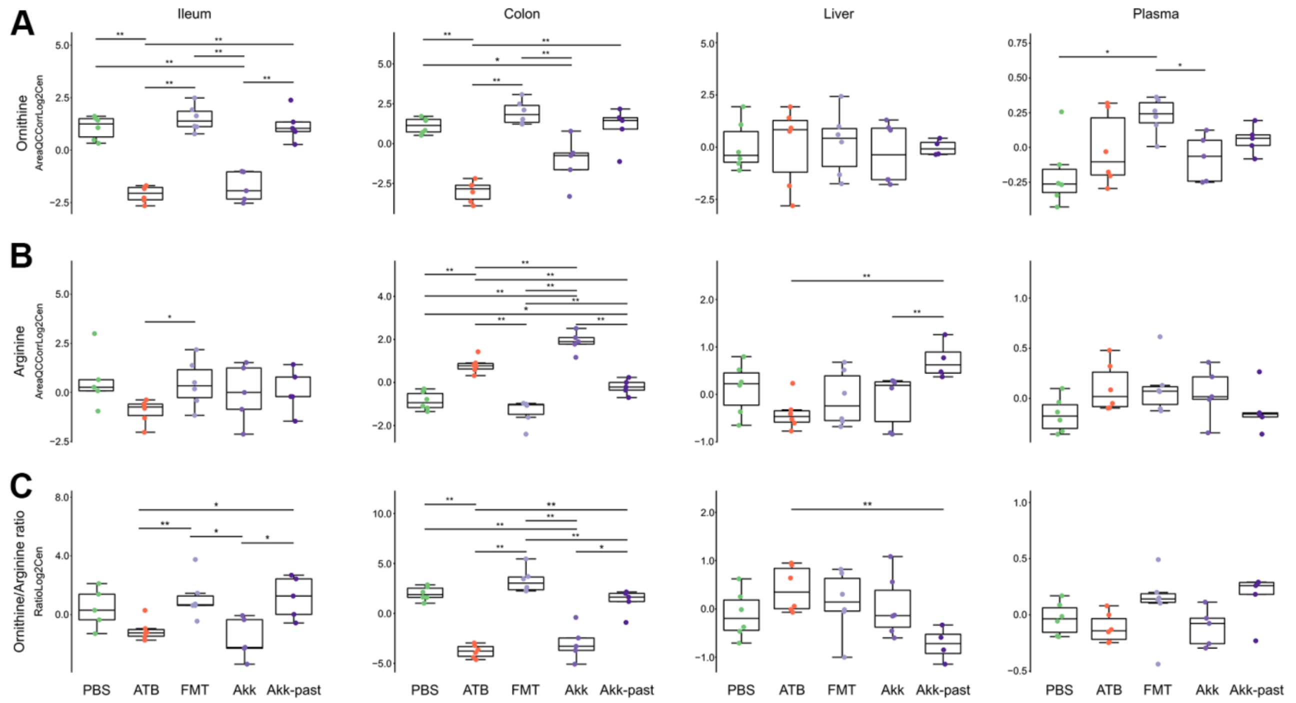

Supplementary Figure 14. Targeted analysis performed on ileum, colon, liver, and plasma samples from Akk- or Akk-past treated mice (versus continuous FMT or ATB, and PBS) allowed the identification of ornithine (**A**) and its precursor, the amino acid arginine (**B**). The relative abundances of these metabolites were used to calculate the ornithine/arginine ratio (**C**) per sample. Statistical differences were determined by non-parametric unpaired Wilcoxon test (Mann-Whitney) for each two-group comparison: * p≤0.05; ** p≤0.01; *** p≤0.001.

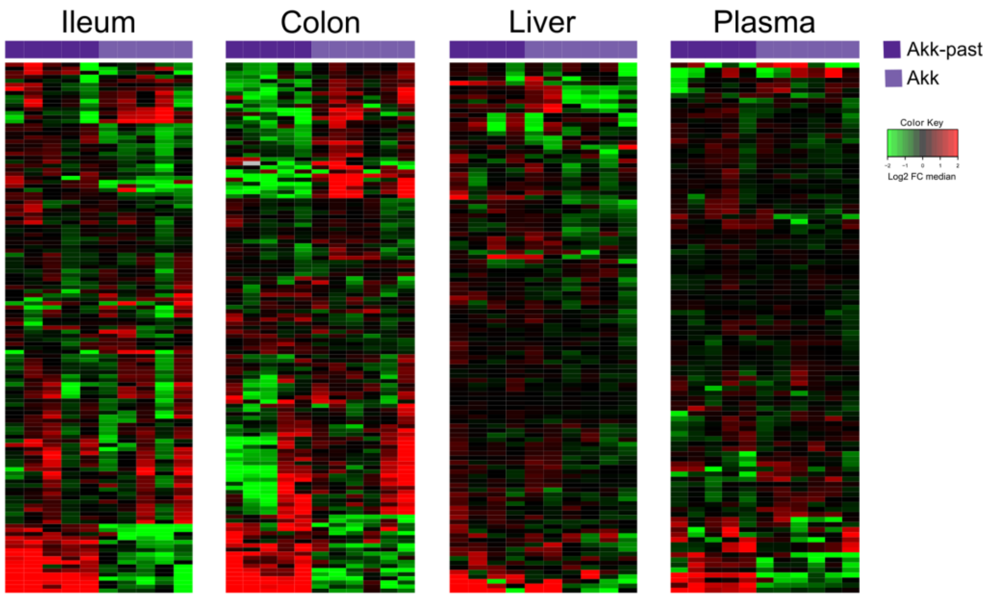

Supplementary Figure 15. Targeted metabolomics analysis was performed on the extracts from ileum, colon, liver, and plasma samples from mice receiving oral gavages with Akk or Akk-past. Changes in metabolites relative abundance are illustrated. Ileum and colon showed the strongest treatment-dependent metabolites variations. Hierarchical clustering (euclidean distance, ward linkage method) of the metabolite abundance is shown. ATB, antibiotics; PBS, phosphate buffer saline; FMT, fecal microbiota transplant; FC, fold change. Note that Supplementary Figures 16 to 19 provide the names of each of the metabolites, for each of the different matrices. The purpose of this figure is to allow for a direct comparison of the amplitude of the metabolic effects of Akk versus Akk-past.

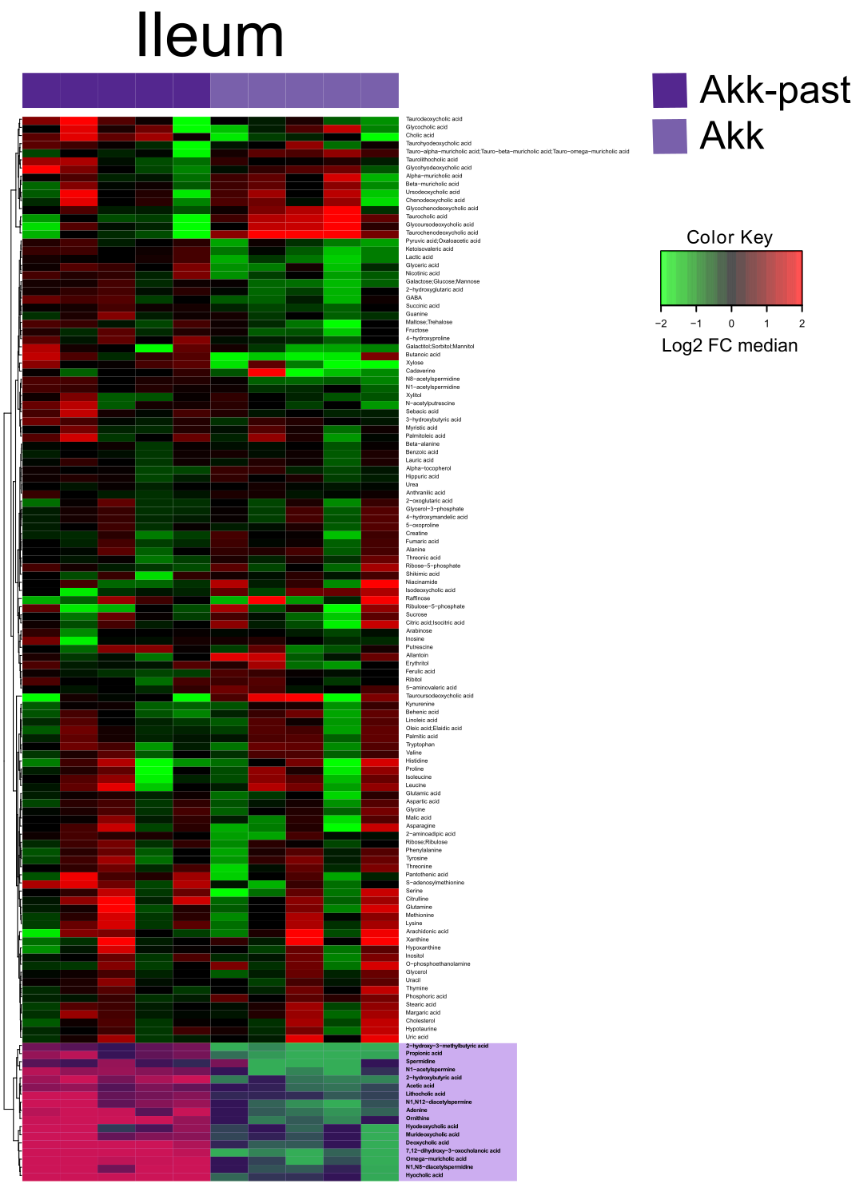

Supplementary Figure 16. Targeted metabolomics analysis was performed on ileum extracts from mice receiving oral gavages with Akk or Akk-past. Changes in metabolites relative abundance are illustrated, with a cluster of metabolites significantly more abundant in Akk-past-treated mice highlighted in purple. Metabolites names and clustering are showed. Hierarchical clustering (euclidean distance, ward linkage method) of the metabolite abundance is shown. ATB, antibiotics; PBS, phosphate buffer saline; FMT, fecal microbiota transplant; FC, fold change.

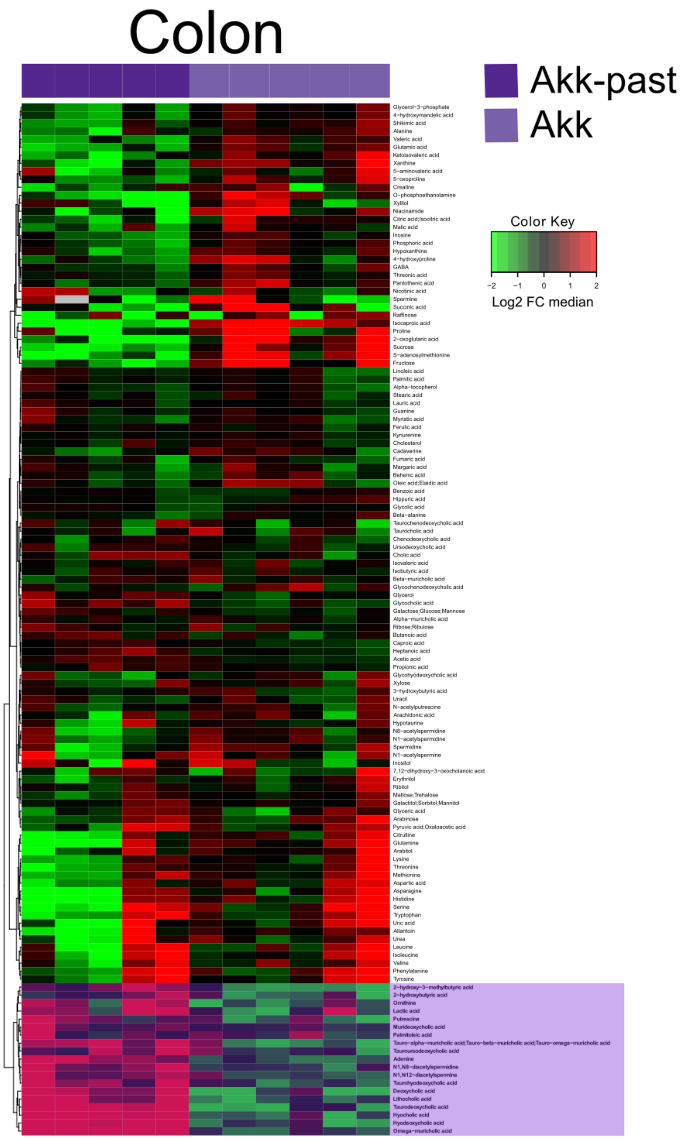

Supplementary Figure 17. Targeted metabolomics analysis was performed on colon extracts from mice receiving oral gavages with Akk or Akk-past. Changes in metabolites relative abundance are illustrated, with a cluster of metabolites significantly more abundant in Akk-past-treated mice highlighted in purple. Metabolites names and clustering are showed. Hierarchical clustering (euclidean distance, ward linkage method) of the metabolite abundance is shown. ATB, antibiotics; PBS, phosphate buffer saline; FMT, fecal microbiota transplant; FC, fold change.

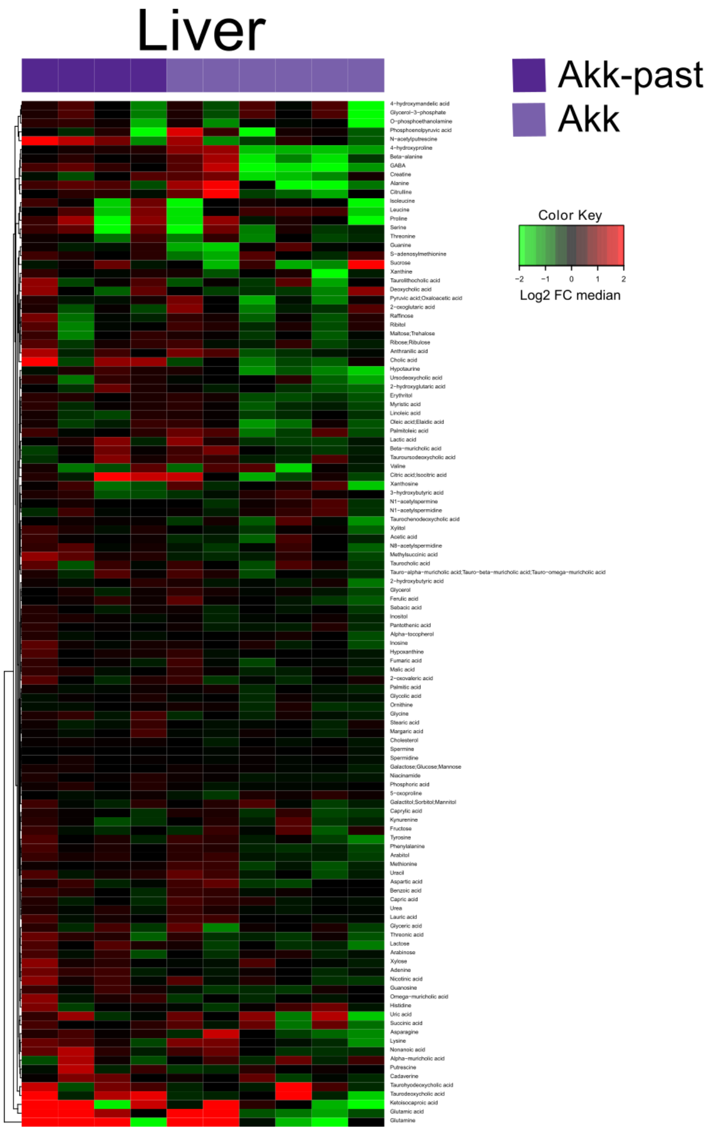

Supplementary Figure 18. Targeted metabolomics analysis was performed on liver extracts from mice receiving oral gavages with Akk or Akk-past. Changes in metabolites relative abundance are illustrated. Metabolites names and clustering are showed. Hierarchical clustering (euclidean distance, ward linkage method) of the metabolite abundance is shown. ATB, antibiotics; PBS, phosphate buffer saline; FMT, fecal microbiota transplant; FC, fold change.

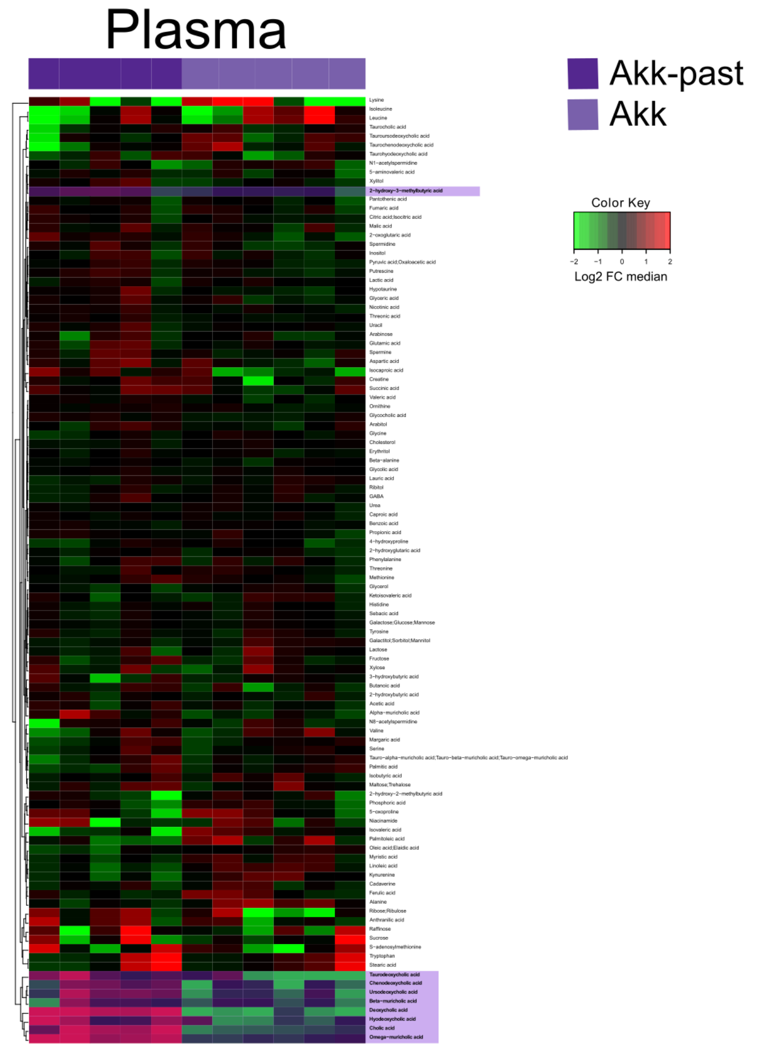

Supplementary Figure 19. Targeted metabolomics analysis was performed on plasma extracts from mice receiving oral gavages with Akk or Akk-past. Changes in metabolites relative abundance are illustrated, with a cluster of metabolites significantly more abundant in Akk-past-treated mice highlighted in purple. Metabolites names and clustering are showed. Hierarchical clustering (euclidean distance, ward linkage method) of the metabolite abundance is shown. ATB, antibiotics; PBS, phosphate buffer saline; FMT, fecal microbiota transplant; FC, fold change.

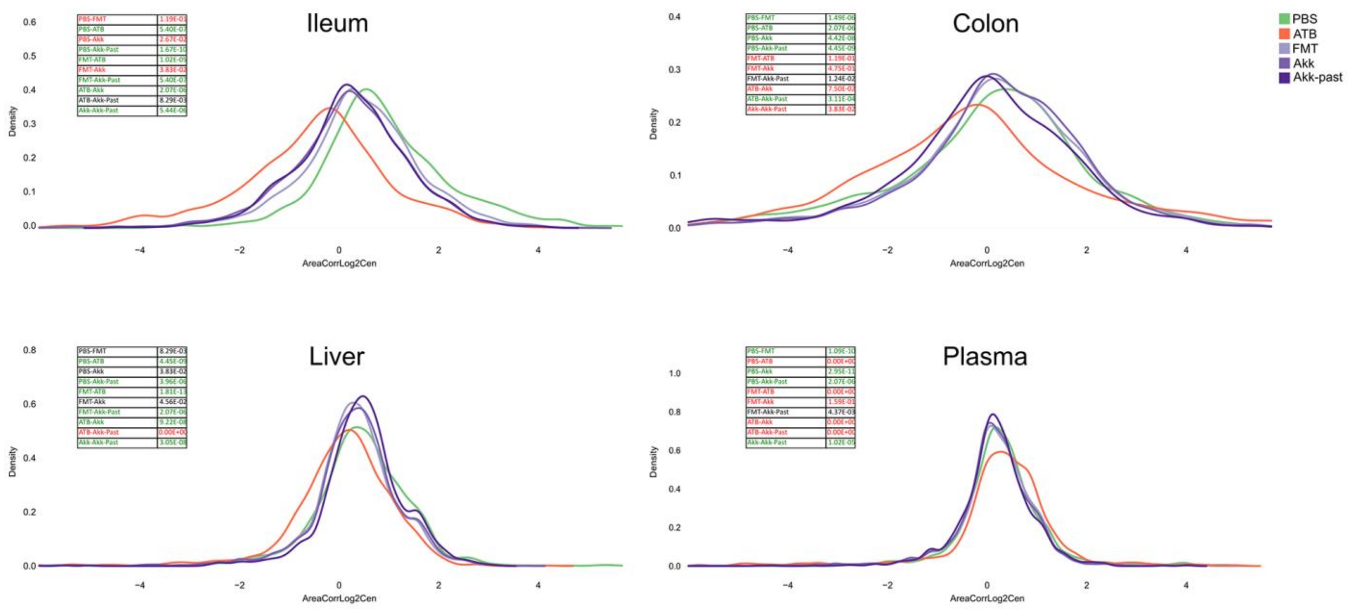

Supplementary Figure 20. Representation in the form of density plot of the distribution of the relative abundance of all metabolites detected using targeted analysis, in the ileum, colon, liver and in the plasma samples from mice treated with FMT, Akk or with Akk-past (versus continuous ATB). For each case, insert shows *p* values obtained with the Kolmogorov–Smirnov non-parametric test, between comparisons. Letters in green, black, and red indicate p≤0.001, p≤0.05 and p>0.05, respectively.
